# Chemonastic Stalked Glands in the Carnivorous Rainbow Plant *Byblis gigantea* LINDL. (Byblidaceae, Lamiales)

**DOI:** 10.3390/ijms231911514

**Published:** 2022-09-29

**Authors:** Simon Poppinga, Noah Knorr, Sebastian Ruppert, Thomas Speck

**Affiliations:** 1 Botanical Garden, Department of Biology, Technical University of Darmstadt, 64287 Darmstadt, Germany; 2 Botanical Garden, University of Freiburg, 79104 Freiburg im Breisgau, Germany; 3 Cluster of Excellence livMatS @ FIT—Freiburg Center for Interactive Materials and Bioinspired Technologies, University of Freiburg, 79110 Freiburg im Breisgau, Germany

**Keywords:** biomechanics, carnivory, functional morphology, prey capture, trichome

## Abstract

Carnivorous rainbow plants (*Byblis*, Byblidaceae, Lamiales) possess sticky flypaper traps for the capture, retention, and digestion of prey (mainly small insects). The trapping system is based on a multitude of millimeter-sized glandular trichomes (also termed stalked glands), which produce adhesive glue drops. For over a century, the trapping system of *Byblis* was considered passive, meaning that no plant movement is involved. Recently, a remarkable discovery was made: the stalked glands of *Byblis* are indeed capable of reacting to chemical (protein) stimuli with slow movement responses. This prompted us to investigate this phenomenon further with a series of experiments on the stimulation, kinematics, actuation, and functional morphology of the stalked glands of cultivated *Byblis gigantea* plants. Measured stalked gland lengths and densities on the trap leaves are similar to the data from the literature. Motion reactions could only be triggered with chemical stimuli, corroborating the prior study on the stalked gland sensitivity. Reaction time (i.e., time from stimulation until the onset of motion) and movement duration are temperature-dependent, which hints towards a tight physiological control of the involved processes. The stalked gland movement, which consist of a sequence of twisting and kinking motions, is rendered possible by the components of the stalk cell wall and is furthermore anatomically and mechanically predetermined by the orientation of cellulose microfibrils in the cell wall. Successive water displacement processes from the stalk cell into the basal cells actuate the movement. The same kinematics could be observed in stalked glands drying in air or submersed in a saturated salt solution. Stimulated and dried stalked glands as well as those from the hypertonic medium were capable of regaining their initial shape by rehydration in water. However, no glue production could be observed afterwards. The long-time overlooked chemonastic movements of stalked glands may help *Byblis* to retain and digest its prey; however, further research is needed to shed light on the ecological characteristics of the rainbow plant’s trapping system.

## 1. Introduction

Carnivorous plants have attracted the interest of scientists since Darwin’s seminal book [1] and more than 800 species have been described until now [2]. They develop highly modified leaves for capture and digestion of animal prey [3,4]. Currently, five main prey capture mechanisms are known: eel-traps, glue traps, pitfall traps, snap traps, and suction traps [5,6]. Traditionally, traps are considered active if they perform motion (e.g., the snap trap of the Venus flytrap), or passive if they do not move (e.g., the pitfall traps of many pitcher plants) [3]. A recent review concludes that traps often possess combinations of motile and non-motile structures and classifies the involved movement processes with respect to the conversion of metabolic energy and the relative timing of energy conversion and motion [7].

Carnivorous plants with glue traps (also termed flypaper traps) catch their prey with a sticky liquid. If a prey animal comes into contact with these glue droplets, it sticks and suffocates [3,4]. Digestive enzymes are secreted and the dissolved nutrients become available to the plant. In several species of sundews (*Drosera* spp.) and butterworts (*Pinguicula* spp.), the whole leaf and/or marginal emergences (i.e., multi-cellular outgrowths often termed tentacles) show bending reactions to either support the capture [8] or the retention and digestion of prey [9,10]. All other carnivorous genera with glue traps (*Byblis, Drosophyllum, Philcoxia, Roridula,* and *Triphyophyllum*) are classically considered to feature non-motile traps [1,3,4,5]. However, a recent study [11] shows that the trapping trichomes (i.e., the stalked glands) of rainbow plants (*Byblis* spp.) are indeed able to perform motion responses to chemical stimulation: the application of fish flake food entailed distinct twisting and bending motions. In contrast, there were no reactions to the application of, e.g., kitchen paper and perlite. These surprising findings prompted us to undertake analyses and experiments to characterize this behavior further, for which we chose *B. gigantea* as a model species.

*Byblis* is the only genus within the carnivorous family Byblidaceae [12,13]. Perennial species (*B. gigantea* and *B. lamellata*) occur exclusively in southwestern Australia and resprout from a partially woody rhizome at the beginning of the rainy season or after a bushfire. Annual species (*B. aquatica, B. filifolia, B. guehoi, B. liniflora, B. pilbarana*, and *B. rorida*) occur in tropical northern Australia, where they grow during the rainy season [13]. *B. liniflora* can also be found in New Guinea. Common to all *Byblis* species is their occurrence on nutrient-poor substrates [12,14,15], which is compensated, as in other carnivorous plants, by prey capture and subsequent digestion and nutrient uptake [3,4,5].

**Figure 1:**
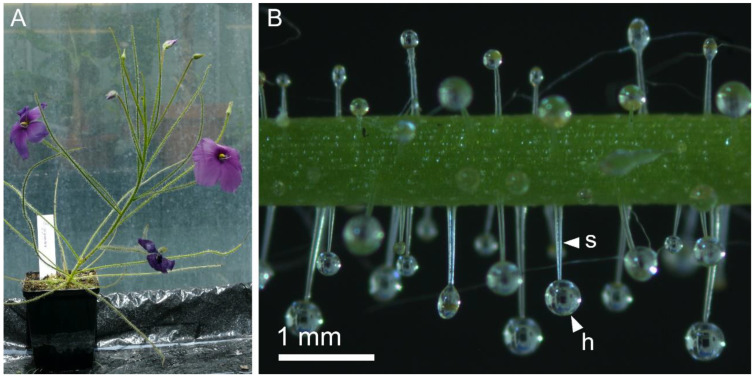
*B. gigantea* in cultivation. (**A**) A flowering plant. (**B**) Detail of a trap leaf. The lower leaf surface shows a higher stalked gland density than the upper surface. The stalk cell (s) and head (h) with a glue drop of a single stalked gland situated on the lower leaf surface are indicated.

The perennial *B. gigantea* occurs in a small area near the city of Perth in Western Australia. It grows in swamps, which are wet for most of the year, often together with many other carnivorous plant species [13,15]. *B. gigantea* can grow as a sub-shrub and reaches heights of up to 50 cm. The flower-bearing shoots are unbranched and carry 10–20 cm long cylindrical leaves [3] (Figure 1A). Except for the petals, all airborne organs are covered with long stalked glands, which produce the trapping glue [16] (Figure 1B), and with short digestive glands for digesting the prey and nutrient uptake [3,17,18]. The density of stalked glands is higher on the lower surfaces of the trapping leaves than on their upper surfaces [11].

Stalked glands consist of a long stalk cell, a neck cell, and a head consisting of 32 and, in rare cases, up to 50 cells. The stalk cell is anchored between four to eight basal cells in the epidermis [19,20]. The short-stalked digestive glands are arranged in rows and situated in epidermal grooves between the stomata on the epidermis. They secrete mucus and digestive enzymes and additionally serve for the uptake of nutrients [3,17,18]. The head of a digestive gland consists of eight cells and sits on a short stalk cell, without the presence of a neck cell. The stalk cell is anchored in the epidermis by two basal cells [19,20].

To gain a better understanding of the trapping system of *B. gigantea*, we conducted a series of descriptive and experimental approaches. This allowed a detailed characterization of the stimulation, kinematics, actuation principle, and functional morphology of the stalked glands.

## 2. Results

### 2.1. Lengths and Densities of Stalked Glands

**Figure 2:**
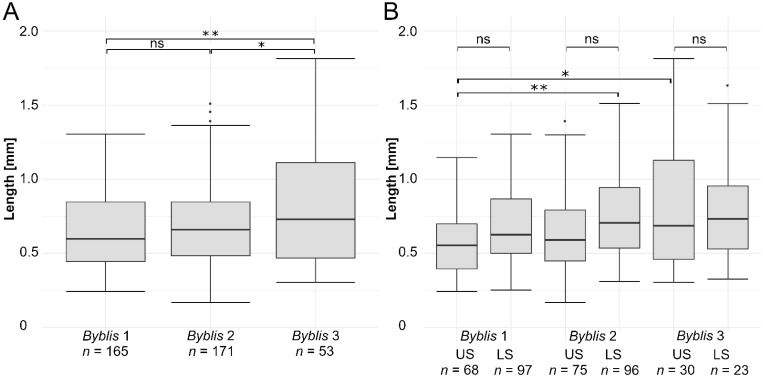
Stalked gland lengths measured on three leaves from three *B. gigantea* plants (*Byblis*-1, *Byblis*-2, *Byblis*-3) and grouped according to the respective plants in (**A**) and according to the respective plants and positions on the leaves (upper and lower surfaces) in (**B**). The asterisks indicate the significance levels (* 0.01 ≤ *p* < 0.05; ** ≙ 0.001 ≤ *p* < 0.01; ns ≙ not significant). The sample sizes (*n*) are indicated. US = upper surface of the respective leaf; LS = lower surface. For clarity, the non-significant results only, between the upper and lower surfaces of the individual leaves, are indicated in (**B**).

The longest stalked gland was 2.74 mm long; the shortest one 0.17 mm. Figure 2A shows boxplots of the measured stalked gland lengths which are grouped according to the respective plants. Stalked glands were 0.64 ± 0.25 mm (minimum = 0.24 mm; maximum = 1.30 mm, *n* = 166) long in *Byblis*-1; 0.70 ± 0.28 mm (minimum = 0.17 mm; maximum = 1.51 mm, *n* = 171) in *Byblis*-2; and 0.97 ± 0.61 mm (minimum = 0.31 mm; maximum = 2.74 mm, *n* = 53) in *Byblis*-3. A significant difference in stalked gland length between *Byblis*-2 and *Byblis*-3 (Kruskal–Wallis Test, χ^2^(2) = 11.708, *p* = 0.044, Pairwise Wilcoxon Rank Sum Test) and a very significant difference between *Byblis*-1 and *Byblis*-3 (Kruskal–Wallis Test, χ^2^(2) = 11.708, *p* = 0.003, Pairwise Wilcoxon Rank Sum Test) were detected.

Figure 2B shows boxplots of the measured stalked gland lengths grouped according to the respective plants and positions on the leaves, i.e., their upper and lower surfaces. Stalked glands on the upper surface of the leaf were 0.58 ± 0.24 mm (minimum = 0.24 mm; maximum = 1.15 mm, *n* = 68) long in *Byblis*-1; 0.63 ± 0.25 mm (minimum = 0.17 mm; maximum = 1.39 mm, *n* = 75) in *Byblis*-2; and 1.05 ± 0.70 mm (minimum = 0.31 mm; maximum = 2.74 mm, *n* = 30) in *Byblis*-3. Stalked glands on the lower surface of the leaf were 0.69 ± 0.24 mm (minimum = 0.25 mm; maximum = 1.30 mm, *n* = 97) long in *Byblis*-1; 0.75 ± 0.29 mm (minimum = 0.31 mm; maximum = 1.51 mm, *n* = 96) in *Byblis*-2; and 0.87 ± 0.46 mm (minimum = 0.33 mm; maximum = 2.16 mm, *n* = 23) in *Byblis*-3. A significant difference in stalked gland length between the upper surface of *Byblis*-1 and the upper surface of *Byblis*-3 (Kruskal–Wallis test, χ^2^(5) = 25.337, *p* = 0.0156, Pairwise Wilcoxon Rank Sum Test) and a very significant difference between the upper surface of *Byblis*-1 and the lower surface of *Byblis*-2 (Kruska–Wallis test, χ^2^(5) = 25.337, *p* = 0.0021, Pairwise Wilcoxon Rank Sum Test) were found. All measured stalked gland lengths are provided in Table S1.

Table 1 indicates the stalked gland densities as ratios between the measured stalked gland numbers on the upper and lower leaf surfaces. The lowest ratio of 0.38 was found on leaf 2 of *Byblis*-1, the highest ratio of 0.81 on leaf 1 of *Byblis*-3. *Byblis*-1 had a mean stalked gland density of 0.55 ± 0.15 (*n* = 3), *Byblis*-2 had a mean stalked gland density of 0.45 ± 0.07 (*n* = 3), and *Byblis*-3 had a mean stalked gland density of 0.51 ± 0.20 (*n* = 4). With an overall mean ratio of 0.51 ± 0.14 (*n* = 10, *Byblis* 1–3), it can be concluded that there are about twice as many stalked glands on the lower leaf surfaces as on the upper surfaces (see also Figure 1B). All counted numbers of stalked glands on the upper and lower surfaces of leaves are provided in Table S2.

### 2.2. Stalked Gland Stimulation, Reaction Time, Movement Duration, and Temperature Dependency

**Figure 3:**
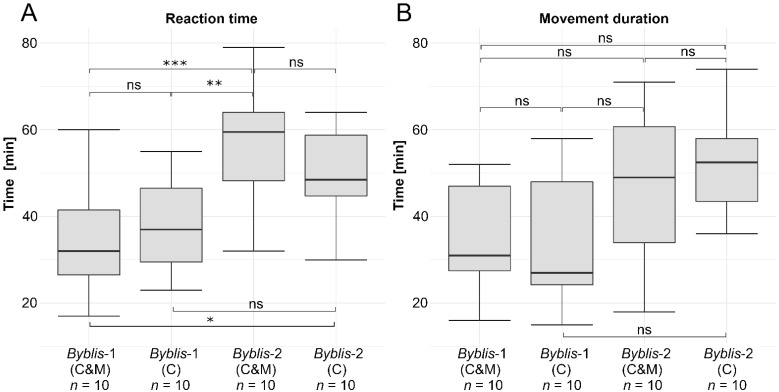
Stalked gland reaction time (**A**) and movement duration (**B**) measured on leaves from two *B. gigantea* plants (*Byblis*-1, *Byblis*-2) and grouped according to the respective stimulation scenarios: pure chemical (C) and combined chemical and mechanical stimulation (C&M). The asterisks indicate the significance levels (* ≙ 0.01 ≤ *p* < 0.05; ** ≙ 0.001 ≤ *p* < 0.01; *** ≙ *p* < 0.001; ns ≙ not significant). The sample sizes (*n*) are indicated.

None of the tested stalked glands showed responses to the pure mechanical stimulation by bursts of consecutive touches with an eyelash to their heads and bases, as well as by the application of Styrofoam beads. In contrast, the pure chemical as well as the combined chemical and mechanical stimulation scenarios triggered movements in all tested stalked glands. Figure 3A shows boxplots of the measured reaction times grouped according to the respective plants and stimulation scenarios, i.e., pure chemical (C) vs. combined chemical and mechanical stimulation (C&M). Reaction times were 37.7 ± 11.1 min (minimum = 23 min; maximum = 55 min, *n* = 10) in *Byblis*-1 (C); 34.2 ± 12.4 min (minimum = 17 min; maximum = 60 min, *n* = 10) in *Byblis*-1 (C&M); 49.8 ± 10.5 min (minimum = 30 min; maximum = 64 min, *n* = 10) in *Byblis*-2 (C); and 57.6 ± 14.7 min (minimum = 32 min; maximum = 79 min, *n* = 10) in *Byblis*-2 (C&M). The shortest reaction time (17 min) was measured in *Byblis*-1 (C&M); the longest one (79 min) in *Byblis*-2 (C&M). A significant difference in reaction times was found between *Byblis*-1 (C&M) and *Byblis*-2 (C) (one-way analysis of variance, F(3.36) = 7.779, *p* = 0.0353206, Tukey HSD post-hoc testing); very significant differences were found between *Byblis*-1 (C) and *Bybils*-2 (C&M) (One-way Analysis of Variance, F(3,36) = 7.779, *p* = 0.0046993, Tukey HSD post-hoc testing); and extremely significant differences were found between *Byblis*-1 (C&M) and *Byblis*-2 (C&M) (One-way Analysis of Variance, F(3,36) = 7.779, *p* = 0.0007705, Tukey HSD post-hoc testing).

Figure 3B shows boxplots of the measured movement duration of stimulated stalked glands. Movement duration were 34.2 ± 16.2 min (minimum = 15 min; maximum = 58 min, *n* = 10) in *Byblis*-1 (C); 35.5 ± 12.3 min (minimum = 16 min; maximum = 52 min, *n* = 10) in *Byblis*-1 (C&M); 51.8 ± 11.5 min (minimum = 36 min; maximum = 74 min, *n* = 53) in *Byblis*-2 (C); and 46.7 ± 18.6 min (minimum = 18 min; maximum = 71 min, *n* = 10) in *Byblis*-2 (C&M). The shortest movement duration (15 min) was measured in *Byblis*-1 (C); the longest (74 min) in *Byblis*-2 I. There are no significant differences between the two tested plants and stimulation scenarios.

During the stimulation tests, fluctuating relative humidity (26.9–55.7%) and temperature (23.2 °C–34.6 °C) were noted. The successfully stimulated stalked glands (i.e., from stimulation scenarios C and C&M) were 0.89 ± 0.24 mm (minimum = 0.30 mm; maximum = 1.32 mm, *n* = 40) long. All measured values for reaction time, movement duration, temperature, relative humidity, and stalked gland lengths are provided in Table S3. The results from the respective correlation tests can be seen in Figure S1. Significant positive correlations were found between the movement duration and the relative humidity for *Byblis*-1 (C&M) (Pearson’s product-moment correlation, r(8) = 0.774, *p* = 0.0085) (Figure S1D); and between the movement duration and the stalked gland length for *Byblis*-1 (C&M) (Pearson’s product-moment correlation, r(8) = 0.676, *p* = 0.032) (Figure S1F). All other tested cases did not correlate significantly.

Stimulation of stalked glands on detached leaf pieces always resulted in movement, even several days after the leaf-piece was cut off. Movie S1 shows the movement evoked by immediate chemical stimulation after detachment of the leaf-piece. Figure S2 shows the movement responses of two stalked glands from the same leaf-piece stimulated after 10 min and after three days and 60 min, respectively.

**Figure 4:**
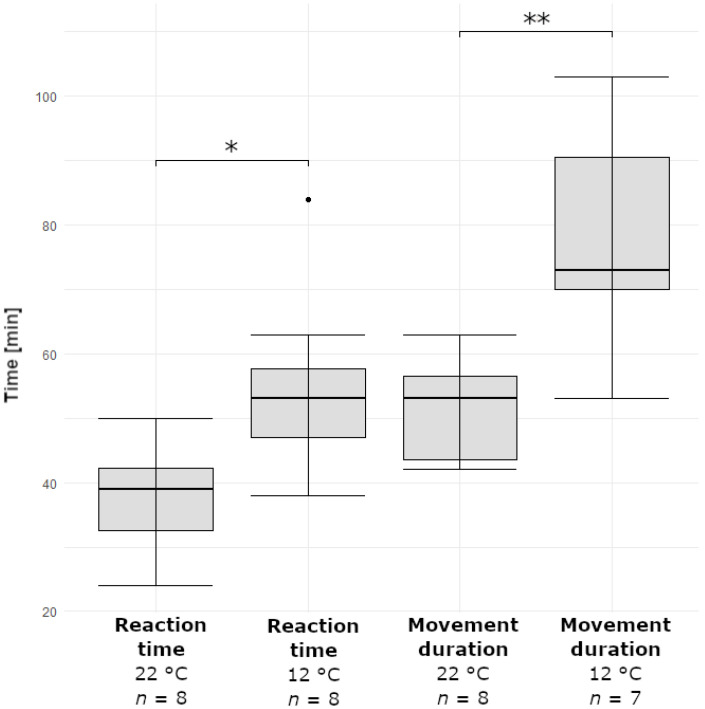
Comparative analyses of reaction time and movement duration of stalked glands triggered in warm (22 °C) and cold (12 °C) temperature regimes. The asterisks indicate the significance levels (* ≙ 0.01 ≤ *p* < 0.05; ** ≙ 0.001 ≤ *p* < 0.01; ns ≙ not significant). The sample sizes (*n*) are indicated.

Figure 4 shows the results of the comparative movement analyses of stalked glands stimulated in warm (22 °C) and cold (12 °C) temperature regimes. Reaction times were 37.6 ± 8.7 min (minimum = 24 min; maximum = 50 min, *n* = 8) in the warm regime and 54.9 ± 14.0 min (minimum = 38 min; maximum = 84 min, *n* = 8) in the cold regime. Movement duration was 51.4 ± 8.0 min (minimum = 42 min; maximum = 63 min, *n* = 8) in the warm regime and 80.0± 16.7 min (minimum = 53 min; maximum = 103 min, *n* = 7) in the cold regime. In summary, the reaction time was significantly longer and the movement duration was very significantly longer in the cold regime. Two incomplete movements (i.e., movements that stopped early) were additionally noted in the cold regime, one of them is exemplarily shown in Movie S2. The reaction time and movement duration measured in the two temperature regimes are provided in Table S4.

### 2.3. Kinematics of Stalked Glands

**Figure 5:**
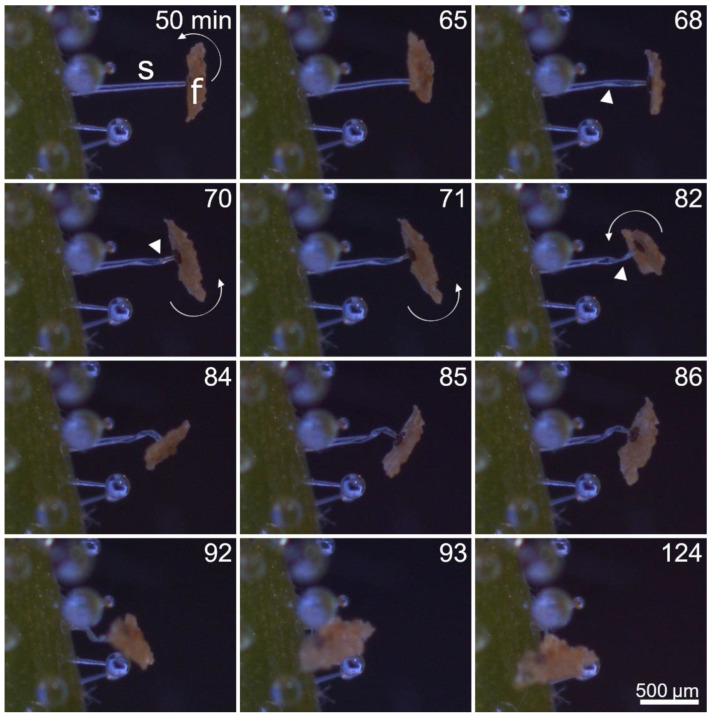
Kinematics of a stalked gland stimulated with a fish food flake (f). 50 min after stimulation, the stalked gland begins to move. Initially, the stalk cell (s) twists, which causes the head to rotate counterclockwise (indicated with fine white arrows). The stalk cell kinks numerous times (indicated by thick white arrows) and finally collapses. Times [min] are indicated. The images are frames from Movie S3.

After pure chemical or combined chemical and mechanical stimulation, the stalked glands typically performed consecutive motion steps consisting of a counter-clockwise twisting motion and a kinking of the stalk cell (Figure 5; Movie S3). The extent of the two steps may vary. In Movie S4, the stalked gland only shows the twisting motion and no kink is formed at any point, whereas in Movie S5 a kink is forming while only a slight preceding twisting motion can be observed. However, each observed motion started with a twisting at the top of the stalk cell. In the cases where the twisting was very prominent, the stalk cell spiraled up like a corkscrew. Both during and after the twisting, kinks may form in the stalk cell, leading to a collapse of the stalked gland to the leaf surface.

Figure 5 presents a representative kinematic pattern of a chemically stimulated stalked gland. The stalk cell begins with the twisting motion 50 min after stimulation, causing the head to rotate. After 68 min, the stalk cell kinks but remains upright and continues to twist. After 82 min, another kink occurs on the stalk cell, causing the head of the stalked gland to tilt (see also minute 84). However, the head continues to rotate. After 86 min, the stalk cell kinks for the third time and the fish food flake is pulled towards the leaf surface. Afterwards, only slight further movements can be observed. One hundred and twenty-four min after stimulation, the stalked gland does not move anymore. In other instances, the respective stalked gland contacted the neighboring structures such as other stalked glands with glue drops, which affected the extent of the motion and sometimes even blocked it.

**Figure 6:**
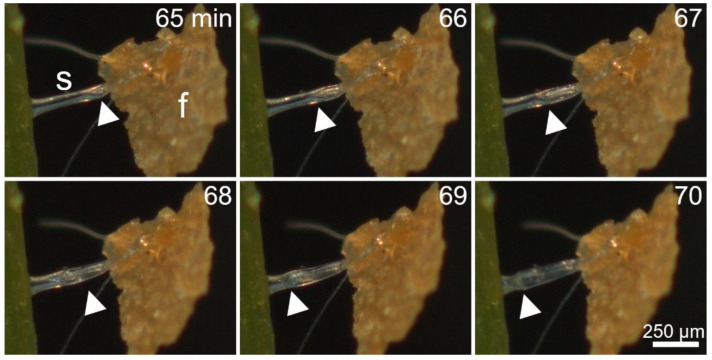
Deformation of the stalk cell (s) after stimulation with a fish food flake (f). The thick white arrows indicate the boundary region between the still cylindrical area and the already flattened area of the stalk cell, which proceeds towards its basal region over the course of time. Times [min] are indicated. The images are frames from Movie S6.

Figure 6 and Movie S6 show details of the deformation of the stalk cell. Sixty-five min after stimulation of the respective stalked gland, the stalk cell kinks for the first time and flattens at this region. The boundary between the still cylindrical area and the already flattened area of the stalk cell proceeds further towards the basal region of the stalked gland over the course of time, which is apparently accompanied by a continuous loss of water from the stalk cell. After 70 min, the stalk cell is almost completely flattened.

The reaction time and movement duration of the six chemically stimulated stalked glands moving in paraffin oil are in the same range to those moving in air (Figure S3). In addition, Movie S7 shows that the kinematics are not different and that the loss of fluid from the stalk cell associated with the movement is not linked to water bubble formation, indicating that no water has been released through the cell wall of the stalk cell to the surrounding medium.

The application of the dead aphid to the head of a stalked gland resulted in the characteristic movement patterns within the typical time scales as reported from the other stimulation experiments using fish food flakes (Figure S4). The struggling of the fruit fly on the trap leaf did not cause any stalked glands to kink or break (Movie S8).

### 2.4. Dehydration and Rehydration of Stalked Glands

**Figure 7:**
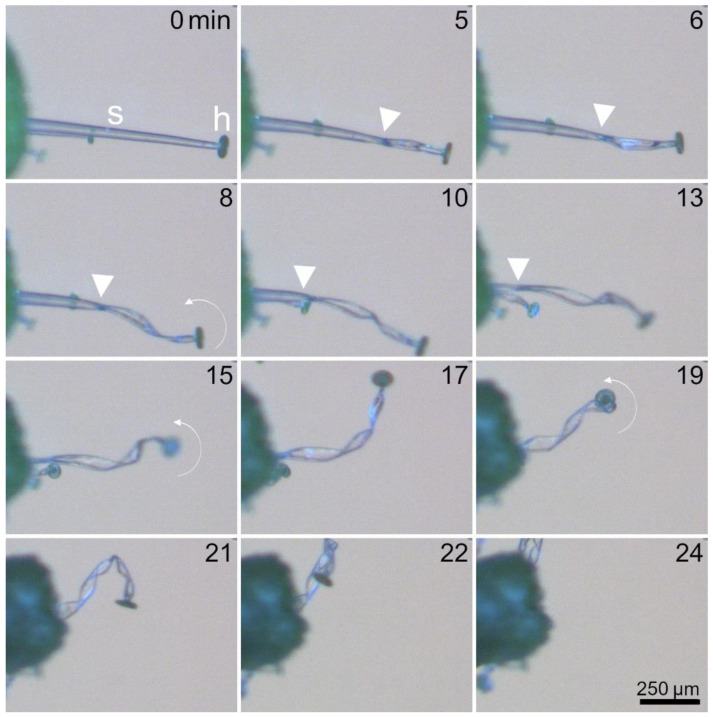
Kinematics of a stalked gland drying in air. The thick white arrows indicate the boundary region between the still cylindrical area and the already flattened area of the stalk cell (s), which proceeds towards the basal region of the stalk cell over the course of time. The head (h) of the stalked gland is indicated. Fine white arrows indicate twisting of the stalk cell and rotation of the head. Times [min] are indicated. The images are frames from Movie S9.

Figure 7 and Movie S9 show the motion patterns of stalked glands drying in air. At the beginning, the stalk cell is fully hydrated and has a cylindrical shape. After five minutes, the first kink forms. The progress of the flattening of the stalk cell can be traced by observing the boundary between the still-cylindrical area and the already flattened area of the stalk cell. Simultaneously with the flattening, the stalked gland undergoes a twisting deformation. After 15 min, the entire stalk cell is flattened, but continues to twist until the head of the stalked gland touches the leaf surface after 24 min. In summary, the kinematics are very similar to those observed in stimulated stalked glands (Figure 5 and Figure 6).

**Figure 8:**
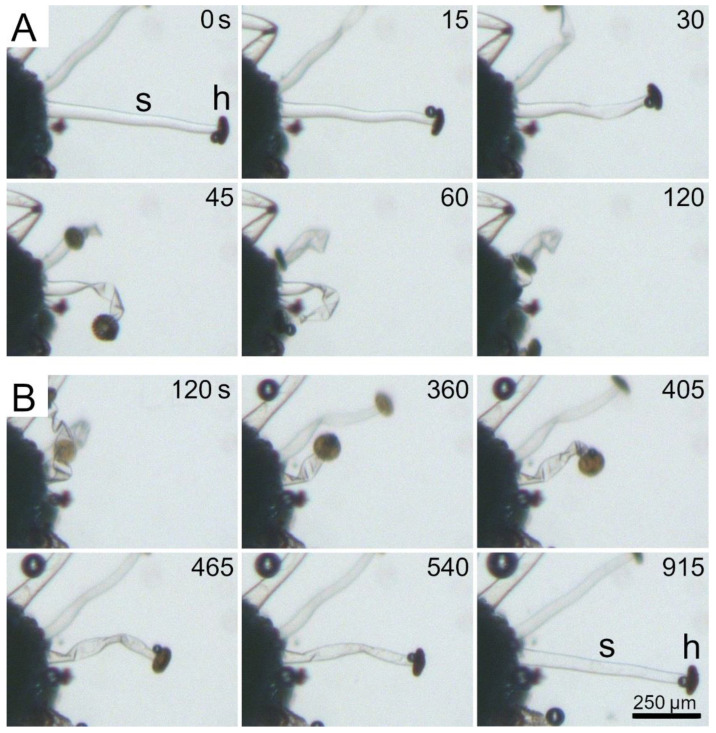
Dehydration and rehydration experiments. (**A**) Dehydration of a stalked gland in a hypertonic medium (saturated NaCl salt solution) and (**B**) its rehydration via immersion in a hypotonic medium (distilled water). The stalk cell (s) and head (h) are indicated. Both movements are basically identical in terms of their general patterns, but in reverse and with individual small differences and very different process times. Times [min] are indicated. The images are frames from Movie S10.

Figure 8A shows the dehydration of a stalked gland via immersion in a saturated salt (NaCl) solution. The stalk cell begins to twist and kink as early as 15 s after addition of the hypertonic medium. The deformation and the motions continue until the stalk cell has completely flattened and the whole stalked gland has collapsed onto the leaf surface. Apart from the different timescales, the observed kinematics are very similar to those observed after stimulation (Figure 5 and Figure 6) and during drying in air (Figure 7). Figure 8B shows the rehydration of the same stalked gland as shown in Figure 8A via immersion in distilled water, which takes much more time than the dehydration. One hundred and twenty seconds after exposure to the hypotonic medium, the shape of the stalked gland has changed only slightly, whereas the movement during dehydration was already complete at this point. Afterwards, the stalked gland performs an un-twisting of its stalk cell, accompanied with rotational motion of its head, until it has regained its fully hydrated state after 915 s. After this shape recovery, the stalked gland morphology cannot be distinguished from its initial shape in Figure 8A. Although the rehydration kinematics are not exactly the reversed dehydration kinematics, the general patterns and sequences are identical. Both dehydration and rehydration processes can also be seen in Movie S10.

The rehydration of a chemically stimulated and collapsed stalked gland in distilled water can be seen in Movie S11. Also here, an untwisting and unbending motion can be recognized until the stalked gland attains its original shape after 112 min. After rehydration, stalked glands were not able to produce glue, in contrast to surrounding, non-stimulated stalked glands (Figure S5).

### 2.5. Functional Morphology of Stalked Glands

#### 2.5.1. General Results

**Figure 9:**
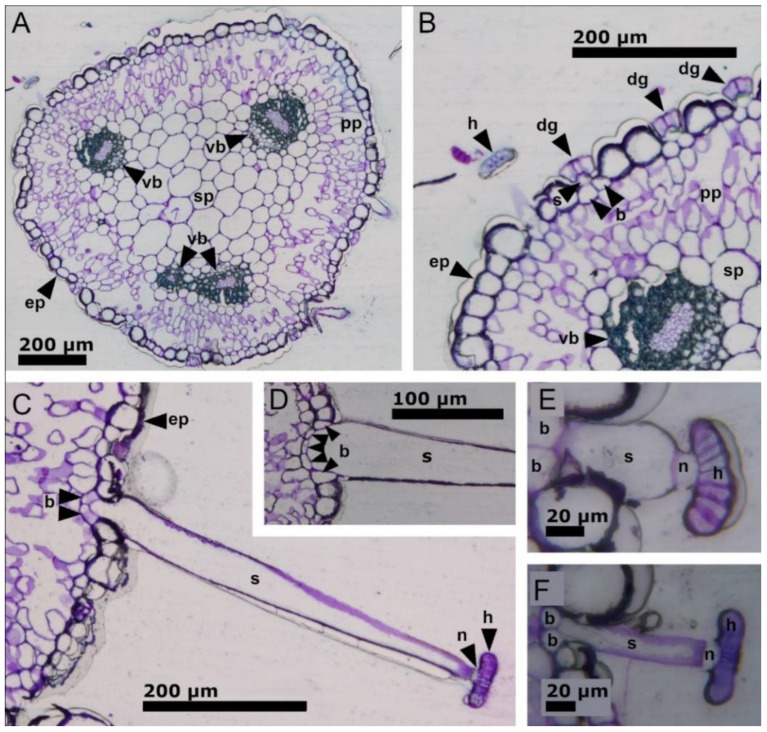
Light microscopy investigations of *B. gigantea* leaves and stalked glands. (**A**) Cross section of a leaf. The vascular bundles, epidermis, palisade parenchyma and spongy parenchyma are visible. (**B**) Detail from (**A**). The digestive glands with basal cells and short stalk cells and a single stalked gland with the head are visible. (**C**–**F**) Individual stalked glands with basal cells (two in **C**,**E**,**F**; five in **D**), long stalk cells, neck cells (which did not stain well with toluidine), and heads. Abbreviations: b = basal cell; dg = digestive gland; ep = epidermis; h = head; n = neck cell; pp = palisade parenchyma; s = stalk cell; sp = spongy parenchyma; and vb = vascular bundle.

Figure 9 presents the results from the light microscopy histological examinations with toluidine staining. All stalked glands shown are non-stimulated. Figure 9A shows a cross section of a leaf, where the vascular bundles, epidermis, palisade parenchyma, and spongy parenchyma are visible. Figure 9B is a detailed view of this section, where the architecture of digestive glands can be recognized. They align with the epidermis and each consists of two basal cells, which are connected to the palisade parenchyma, a short stalk cell, and the head. A cut-off stalked gland with a recognizable head is also present. Figure 9C–F shows sections of differently sized stalked glands, which are each composed of two (Figure 9C,E,F) or five (Figure 9D) basal cells, a long stalk cell, a neck cell, and the head. With a length of only 0.06 mm, the single stalked gland in Figure 9E was exceptionally small. All other lengths of the stalked glands in this study fitted well into the range as indicated in Section 2.1. The basal cells are situated between the epidermal cells. The neck cells did not stain well with toluidine (Figure 9C,E,F).

**Figure 10:**
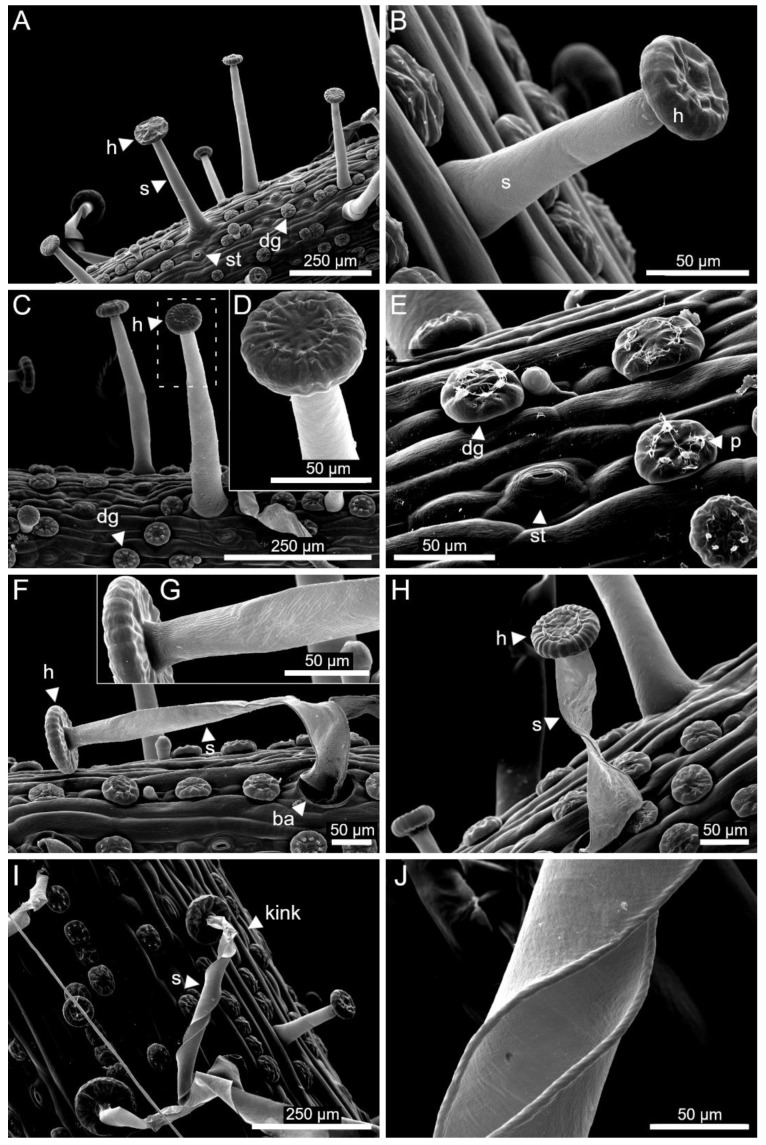
SEM images of critical point-dried *B. gigantea* leaves with non-stimulated and stimulated stalked glands. (**A**–**D**) Leaf surfaces with stomata and digestive glands and non-stimulated stalked glands with stalk cells and heads. (**D**) is a detailed view of the stalked gland framed with a dashed white line in (**C**). (**E**) is a detailed view of the leaf surface with stomata and digestive glands. On the digestive glands, pores with mucus residues are visible (thick white arrow). (**F**–**I**) Leaf surfaces with stimulated stalked glands. The respective stalk cells are flattened from their apical to their basal regions and have kinked and twisted to different degrees. (**G**) is a detailed view of the stalked gland in (**H**). (**J**) is a detailed view of a flattened and twisted stalk cell.

The results from the SEM examinations of critical point-dried leaf fragments with stalked glands can be seen in Figure 10. The stalked glands in Figure 10A–D were non-stimulated and show cylindrical stalk cells and heads with irregular furrowing. Stomata and digestive glands occur on the leaf surfaces. In Figure 10E, pores with mucus residues can be seen on the digestive glands. The stalked glands in Figure 10F–J were stimulated prior to the SEM investigations and their stalk cells appear to be shrunken and folded. The stalk cell of the stalked gland in Figure 10F,G is strongly bent so that the head almost touches the leaf surface. At the shrunken and folded stalked gland base, the severe volume loss that affected the stalk cell during drying is visible. Figure 10G shows in detail that the stalk cell region closely below the head is not flattened, which contrasts with the rest of the stalk cell. The stalk cell of the stalked gland in Figure 10H shows a twist, but has remained nearly completely upright. The stalk cell of the stalked gland in Figure 10I shows numerous twists and has bent towards the leaf surface. Again, a different folding pattern can be observed in the upper part of the stalk cell. While the rest of the stalk cell is twisted, the upper region has folded and kinked. Details of a flattened and twisted stalk cell can be seen in Figure 10J, where the opposing cell walls lie almost on top of each other.

#### 2.5.2. Cellulose Microfibril Angles in Stalk Cells

**Figure 11:**
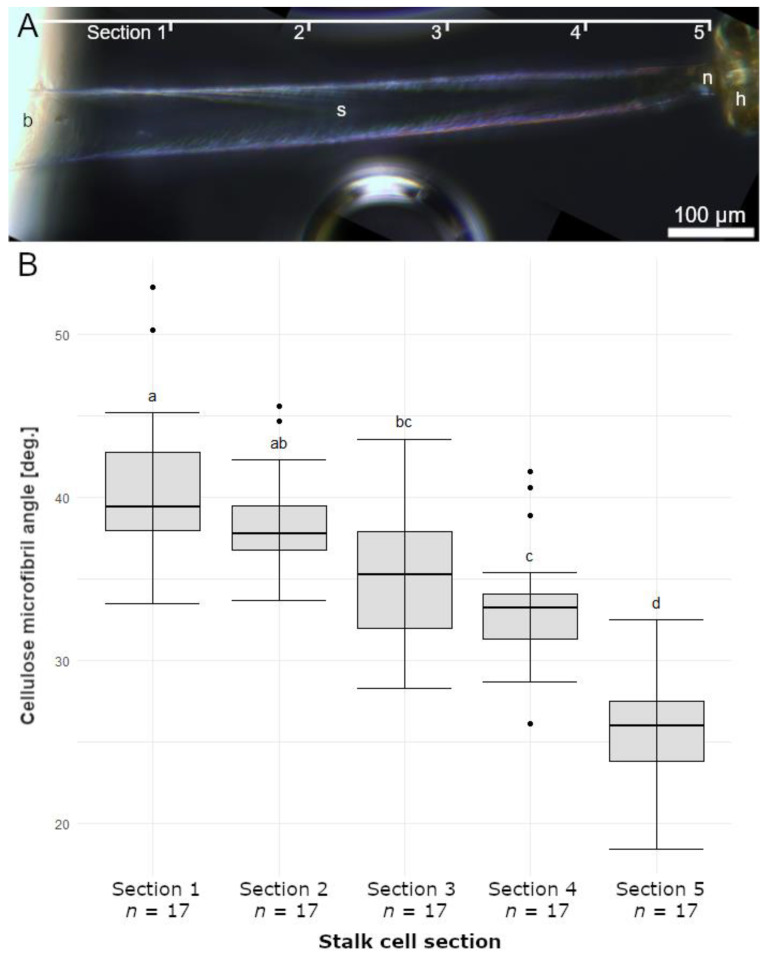
Cellulose microfibril angles in stalk cells. (**A**) Stalked gland divided into equally sized Sections 1—5. Section 1 represents the area at the base (b) of the stalk cell (s) near the trap leaf, whereas Section 5 is located directly below the head (h) and the neck cell (n). (**B**) The cellulose microfibril angles determined in relation to the long axis of each section are shown as boxplots. The cellulose microfibril angles become more acute from the base to the head of the stalked gland. The sample sizes (*n*) are indicated.

Figure 11A shows an investigated stalked gland divided into equally sized sections. Section 1 represents the area at the base of the stalked gland near the trap leaf, whereas Section 5 is located directly below the head and the neck cell.

In Figure 11B, the measured cellulose microfibril angles grouped according to the respective sections are shown as boxplots. Section 1 is characterized by cellulose microfibril angels of 40.9 ± 4.8° (minimum = 33.5°; maximum = 52.9°, *n* = 17); Section 2 of 38.4 ± 3.2° (minimum = 33.7°; maximum = 45.6°, *n* = 17); Section 3 of 35.19 ± 4.3° (minimum = 28.3°; maximum = 43.6°, *n* = 17); Section 4 of 33.5 ± 3.8° (minimum = 26.1°; maximum = 41.6°, *n* = 17); and Section 5 of 25.7 ± 3.2° (minimum = 18.4°; maximum = 32.5°, *n* = 17). Altogether, the cellulose microfibril angles become more acute from the base to the head of the stalked gland, that is, they become more aligned, or more parallel with the longitudinal axis of the stalk cell. The angles of all non-adjacent sections, as well as those of the directly adjacent Sections 4 and 5, differ significantly. All measured angles at the different stalked gland sections are presented in Table S5.

#### 2.5.3. Histological Staining of Stalk Cells

**Figure 12:**
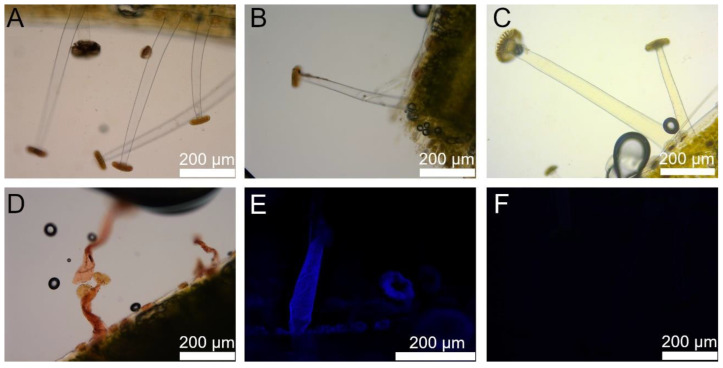
Results from the histological staining approaches. Image (**A**) shows unstained stalked glands as controls. Image (**B**) shows the negative result of ruthenium red staining, (**C**) the negative result of phloroglucinol staining, (**D**) the positive result of Sudan IV staining, and (**E**) the positive result of Calcofluor white staining with the corresponding negative control in (**F**).

Figure 12 shows the results of the histological staining analyses, with Figure 12A depicting unstained stalked glands as controls. Both the ruthenium red (Figure 12B) and phloroglucinol staining (Figure 12C) were negative. Thus, neither pectin nor lignin is significantly present in the cell wall of the stalk cells. The Sudan IV staining (Figure 12D) was positive, indicating the presence of cutin in the cell wall. Here, the staining agent caused a dehydration of the stalked glands, however, no turgescent trichome was necessary for evaluation. Not surprisingly, the positive Calcofluor White staining (Figure 12E) highlights cellulose in the cell wall. Figure 12F shows the negative control of the Calcofluor White staining approach.

## 3. Discussion

### 3.1. Stalked Gland Lengths and Densities

Stalked gland lengths measured in this study generally agree with reports from the existing literature [21]. Significant differences in stalked gland lengths of *Byblis*-3 in comparison to *Byblis*-2 and very significant differences of *Byblis*-3 in comparison to *Byblis*-1 were observed. Possible explanations could be that the differences are due to morphological plasticity and/or due to different ages of the plants tested, which were initially purchased from a commercial supplier. Also of note in this regard is the difference in sample size, which is only one-third as large in *Byblis*-3 as in the other two individuals. An exceptionally small stalked gland (length: 0.06 mm) was additionally discovered during the histological investigations. Prior research shows that in another flypaper carnivorous plant, *Roridula gorgonias* (Roridulaceae), differently sized trapping trichomes (in combination with the different stickiness of the secreted glue) constitute a multi-hierarchical capture system, with short and stiff trichomes secreting a very sticky glue being adapted to strong, long-term adherence to prey insects, and with long and more flexible trichomes producing a less sticky glue, which is responsible for initial trapping and entanglement of prey [22]. It remains to be investigated whether this is also the case for *Byblis*.

Regarding the comparison of lengths of stalked glands from upper and lower leaf surfaces, significant differences were only found between the upper surface of *Byblis*-1 and the upper surface of *Byblis*-3 and very significant differences were only found between the upper surface of *Byblis*-1 and the lower surface of *Byblis*-2. All other comparisons between the upper and lower surfaces of individual leaves and also between the leaves showed no significant differences. Hence, stalked gland length does not apparently vary within the respective positions on individual leaves, but there might be differences when comparing different plants. Generally, stalked gland lengths did not vary much in our cultivated specimen. Future approaches could perform extensive morphometric comparisons between individual plants and populations from different natural locations to evaluate if and, if yes, why stalked gland length varies.

All plants tested had more stalked glands on their lower leaf surfaces than on their upper surfaces. The exact ratios vary from leaf to leaf (Table 1). Leaf 1 of *Byblis*-3, for example, had a ratio of 0.81, which indicates only a weak difference in stalked gland number, whereas leaf 2 of *Byblis*-1 had a ratio of 0.38, meaning that almost three times as many stalked glands were counted on the lower surface as on the upper surface. In our tests there were, on average, half as many stalked glands on the upper leaf surfaces as on the lower surfaces. The reason for this discrepancy in stalked gland density might be based on the actual prey capture scenario. Species of *Byblis* effectively capture small-to medium-sized flying insects [3,11,23], similar to many co-occurring carnivorous sundews (*Drosera* spp.) [24]. The trapping leaves of *B. gigantea* often have an erect posture (Figure 1) so that the lower surfaces of the leaves face outward and presumably capture more prey items than the respective upper leaf surfaces [11,25]. When the prey stick to the upper surface of the trapping leaves, their bodies would be pulled towards the leaf surface due to gravity, making escape more difficult. On the lower surface, their bodies would be pulled away from the trap leaf. A higher number of sticky stalked glands could probably prevent such nutrient losses due to a higher contact area between the trapping glands and the prey. Further research could deal with actual prey-capture experiments to record the behavior of the prey on different locations on the trap leaf, and in response to the mechanical properties of the glue secreted from the stalked glands‘ heads. Prior studies showed that trap mucilage in *Nepenthes* [26] and *Drosera* [27] have viscoelastic properties, which enhances their trapping effectiveness.

### 3.2. Stalked Gland Stimulation, Kinematics, and Actuation

None of the purely mechanical stimuli applied to the heads and bases of stalked glands resulted in movement. When comparing the measured reaction time and movement duration, differences between the purely chemical and the combined chemical and mechanical stimulation scenarios could only be noted between the different tested plants, but not within each tested plant. Therefore, our results corroborate the initial assessment in [11] that the movement of stalked glands is triggered purely chemically. This makes *B. gigantea* the only known strictly chemonastic motile flypaper carnivore, since mechanical triggering is an important component of the sensory systems of both butterworts (*Pinguicula* spp.) and sundews (*Drosera* spp.) [3,6,28].

Fish flake food was used for both the chemical and chemical-mechanical stimulation scenarios as previously reported in [11]. The size of the flake fragments varied between the experimental trials to some extent, however, since the stalked gland kinematics were fairly uniform, we may speculate that flake size had no influence on the movements. Since we took great care to place the flakes on the glue drops without directly touching the stalked gland head, we may assume that there was no (significant) mechanical stimulus involved.

One stalked gland was successfully stimulated with a dead (but otherwise intact) aphid during our experiments, which hints towards a very fine-tuned and precise sensory system. The resulting time scales of motion were in the same regime as those during the other stimulation experiments using fish food flakes.

The results furthermore show that the stalked glands still reacted to chemical stimuli with typical movements even on the detached leaf pieces. This indicates that stalked gland sensitivity and movement are based on local processes, which are presumably not linked to the systemic involvement of the whole plant. Rather, it seems to be crucial that the surrounding tissue is sufficiently hydrated and that desiccation of the leaf-piece does not occur. If these conditions are met, it is possible to conduct experiments over several days investigating the stalked gland movement, without the need for the entire plant. This offers significant methodological freedom for future investigations.

The sensory system of flypaper traps is very well studied in sundews (*Drosera*, *D. capensis* in particular) [9,10,29]. Here, the involved signaling pathways rely on jasmonic acid and are hypothesized to stem from a herbivore defense reaction. However, this pathway could so far only be detected in the trap systems of carnivorous plants from the order Caryophyllales, but not in butterworts (*Pinguicula*), which, like *Byblis*, belong to the Lamiales [30]. Their signal cascade processes which are involved in recognizing prey are, hence, interesting topics for future studies.

The overall movement of the stalked gland consists of a series of twisting and kinking motions of the stalk cell, both of which can vary in extent. In accordance with [11] we may speculate that these motions aid in retaining stuck prey and are for pulling it towards the leaf surface, where the digestive glands are located. This probably enhances the digestion of the prey and the uptake of nutrients. In agreement with [11], we did not witness the concurrent movement of the neighboring stalked glands; hence, the signaling cascade leading to stalked gland movement does not apparently “alarm” the neighboring stalked glands.

According to our correlation analyses, it can be hypothesized that environmental conditions (relative humidity and temperature) and stalk lengths have no, or only weak, effects on stalked gland reaction time and movement duration. However, the possible influences were investigated only with stalked glands from upper leaf surfaces. The influence of the position of the stalked glands on their motion remains, therefore, possible.

According to our observations, the motion actuation involves water displacement from the stalk cell towards the basal cells, beginning at the apical part of the stalk cell near the head (Figure 6). The water displacement is most presumably associated with cohesive pull in the stalk cell, which, in combination with mechanical stresses generated by the deforming cylindrical stalk cell wall caused by diffusive water loss, may drive the observed motion. The water displacement in the direction towards the stalked glands‘ bases basically excludes a possible hygroscopic effect of the fish flake food, which, accordingly, has no direct physical-chemical influence on the movement actuation. Despite the fact that the duration and speed of water-driven plant motion strongly relies on the dimension of the motile structure the water has to pass through [31], stalked gland length did not have an influence on reaction time and movement duration in our experiments, except for one case (Figure S1F). The reason for this remains to be investigated and we can only speculate that other parameters, like temperature and the general vigor of the plant, have a greater impact than their dimensions on stalked gland movement. The comparative analyses of reaction time and movement duration of stalked glands in different temperature regimes (Figure 4) revealed that the movement is indeed strongly dependent on physiological processes. From this result, a purely physical actuation scenario similar to the coiling of stork’s bill (*Erodium*) awns [32] can be excluded with certainty.

The dislocation of the boundary region between the cylindrical (still water-filled) area and the flattened (already water-free) area of the stalk cell during movement could be observed in both stimulated stalked glands as well as in the stalked glands drying in air (Figure 6 and Figure 7). The general kinematics were identical in both cases. This points towards the fact that the identical movement and water displacement in the direction of the leaf can also be evoked by pure physical means, i.e., a low environmental humidity. Probably, *B. gigantea* is able to relocate water from the stalked glands into the leaves and stems to guarantee its survival during dry periods. Since the twisting and buckling processes were also visible during the water withdrawal by the hypertonic medium, we may speculate that these deformation processes are morphologically predetermined by the fine architecture of the stalk cell. This is further corroborated by the fact that, after the rehydration of previously experimentally dehydrated as well as stimulated stalked glands, no morphological differences from their initial state could be detected. This points towards the fact that apparently no permanent anatomical or ultrastructural changes in the stalk cell occur during movement and that water displacement and the resulting cohesive force are its only driving force. Future experiments including, e.g., transmission electron microscopy could identify the fine architecture of desiccated stalk cells for further characterization of their structural integrity.

From the analyses of stalked glands in paraffin oil it can be concluded that no water is released via the stalk cell wall into the environment during the entire movement. This should otherwise be clearly visible as small water droplets in the surrounding paraffin oil. This further corroborates the assumption that the stalked gland movement is driven by the removal of water into the basal cells and it also explains why the movement starts at the stalked gland head and progresses further to the base as the water removal proceeds. In addition, this result suggests that signaling pathways between the head (i.e., the region of stimulus reception), the stalk cell (i.e., the effector cell), and probably also the basal cells of the stalked gland underlie the stimulus response and movement (cf. [11]). There is a high structural similarity between the stalked glands found on *Byblis* and the stalked glands of closely related butterworts (*Pinguicula* spp.) [33], and it would be interesting to investigate whether similar mechanisms of stimulation and actuation exist.

Once the stalked glands have moved and are rehydrated, they are not capable of forming the adhesive glue again. Accordingly, it can be assumed that the stalked glands dehydrate and die upon complete movement and that artificial rehydration has a purely physical effect. In [11] it is noted that many *Byblis* species are annual plants for which multiple-use, more energetically costly stalked glands are probably not worthwhile because a large number of trichomes are formed anyway during leaf development. Furthermore, it was observed in our study with cultivated plants that the chitinous exoskeletons of trapped insects adhered permanently to the leaf surface and were never shed. Even if the stalked glands could regenerate on their own and become sensitive to prey again, the exoskeletons would stick to their heads and possibly render them non-functional.

### 3.3. Functional Morphology of Stalked Glands

Generally, stalked glands are epidermal structures with secretory functions [34]. They occur on 20–30% of known vascular plants and have been observed on all aerial organs [35]. Their function is primarily to protect against biotic and abiotic factors, such as herbivorous insects [36], UV radiation [37], extreme temperature [38], and drought [39]. Another function of stalked glands represents the formation of adhesive traps of various carnivorous plants [40], as described here in *Byblis*. Due to the wide range of operational areas, it is safe to say that the individual trichome types possess fine-tuned functional–morphological adaptations to their tasks. Regarding their basic architecture, the stalked glands of *Byblis*, which are purely epidermal outgrowths without direct connection to the conducting bundle system [20], are different to the glue tentacles of other carnivorous plants, e.g., sundews (*Drosera*). These tentacles constitute glandular emergences, which are traversed by conducting bundle strands [41]. However, both stalked glands and emergences produce sticky secretions that serve to capture prey; therefore, they are very similar in function. The stalked glands of *B. gigantea* are apparently mechanically quite stable and cannot be damaged even by struggling large and strong prey, such as fruit flies, as observed in our study.

The cylindrical leaf of *B. gigantea* is enveloped by the epidermis and contains spongy and palisade parenchyma. Stalked glands, stomata, and the small digestive glands are distributed over the whole leaf surface (Figure 9A,B). The stomata are slightly elevated above the epidermis, which is probably an adaptation to avoid being clogged by the secretions of the digestive glands. The stalked glands may have different numbers of basal cells (Figure 9C–F). Their neck cells could not be stained with toluidine in our histological analyses. A possible explanation for this could be an endodermic suberin incrustation, which otherwise regulates the transport of substances between the leaf and the gland cells [18].

We postulate that the cellulose microfibrils determine the water loss-driven movement of the trichomes. A similar functional–morphological relationship was shown in the attachment system of English ivy (*Hedera helix*) [42]. The root hairs studied there, as well as the stalk cells of the trichomes on *B. gigantea*, are unicellular structures that change shape by fluid withdrawal in a morphologically and anatomically predetermined manner due to the spatial arrangement of the cellulose microfibrils present in the cell wall. As seen well in Figure 11, the angles of the cellulose microfibrils decrease in relation to the central axis of the stalked gland towards its head. This gradient in angles of the cellulose microfibrils provides an explanation for the observed stalked gland movement patterns. Since it involves fluid transport from the head to the base, the stiffening cellulose microfibrils dictate the subsequent deformation patterns in each section. Accordingly, the typical rotational motion of the trichome head at the beginning of the movement can be attributed to the shallow angles that are oriented nearly parallel to the longitudinal axis of the stalked gland. Due to this orientation the stalked gland does not bend and only the head rotates (see the different twisting and folding regions in Figure 10H). Subsequently, increasingly stronger folding movements occur. This also fits to the increasing angles of the cellulose microfibrils, which deviate more and more from the central axis in the direction of the trichome base. The more that these deviate, the more the stalked gland can buckle. This might also explain the observation of fine spiral patterns [1,3], later termed “spiral primary thickenings” [16], on the outer surfaces of stalk cells, which we assume to appear when the stalk cell is not fully turgescent. We can only speculate that the micro-morphological fine-tuning of stalked glands and their complex movement sequences has a selective advantage during prey capture and retention.

The detection of cellulose in the stalk cells with Calcofluor white (Figure 12E) was primarily performed as a control experiment, since the cellulose microfibrils found in the cell walls of the stalked glands are decisive for the rotational and folding movement of the trichomes, as explained above. Cutin, a biopolyester that forms the main component of the plant cuticle, could be detected by means of a positive Sudan IV staining (Figure 12D). The main function of cutin is to prevent the loss of water [43]. Very likely, cutin primarily fulfills this function also in the stalked glands of *B. gigantea*, since their movements exhibit strong dependencies on the fluid content of their stalk cells. Accordingly, it is of high importance for the plant in the context of trap functioning to be able to regulate the water content of the stalk cells in a precise manner and to prevent a passive and uncontrolled water release through evaporation. We therefore assume that the “refilling” of the stalk cell during rehydration follows a pathway of water through the basal and head /neck cells. Furthermore, the stalk cells tested negative for lignin (Figure 12C) and pectin (Figure 12B). These results can be explained by the properties of lignin and pectin and their effect on the cell wall. Hydrophobic lignin performs a stabilizing and mechanical supporting function when incorporated into the cell walls [44]. Thus, lignification of the stalk cells would be in strong contrast to their observed twisting and folding movements. Pectins are complex polysaccharides that can perform various functions [45]. The cell walls of certain trichomes on beech leaves (*Fagus sylvatica*) contain pectins, but no cutin, which serve the uptake of water from the environment [46]. The hygroscopic properties of pectins would negatively interfere with the apparently highly controlled fluid content of the stalk cell of stalked glands in *B. gigantea*. The opposite is true for the cutin content. The results of these measurements clearly illustrate how different components of the cell wall can dictate their properties and thus also the function of the respective trichomes.

### 3.4. Conclusion and Outlook

Our study on *B. gigantea* complements the initial comparative work presented in [11]. The chemonastic movement of stalked glands appears to be under tight physiological control. It can be reversed by pure physical means without the ability of the stalked glands to regain physiological sensitivity. Future approaches could consider performing interspecific comparative analyses to gain insights into possible differences in the trap architecture and function among rainbow plants. The trapping system of *Byblis* is generally poorly studied compared to other genera of carnivorous plants [5]. Accurate and comparative analyses of the prey spectra, the role of commensals [11], the rheological properties of the trapping glue, and the signaling substances and pathways involved in the chemical stimulation and movement initiation are interesting topics for future studies.

## 4. Materials and Methods

### 4.1. Plant Material

*B. gigantea* plants were initially purchased from a commercial supplier (Gartenbau Thomas Carow, Nüdlingen, Germany) and cultivated in the temperate greenhouses of the Botanical Gardens of Freiburg and of the Technical University of Darmstadt according to their requirements.

### 4.2. Lengths and Densities of Stalked Glands

#### 4.2.1. Length Measurements

Length measurements of stalked glands were conducted on healthy leaves from three *B. gigantea* plants (five leaves from *Byblis*-1, seven from *Byblis*-2, and five from *Byblis*-3). Lateral images were taken in the middle, as well as 1 cm to the right and left of the middle of each leaf using a stereo macroscope (M420, Wild, Heerbrugg, Switzerland) equipped with a ColorViewII digital camera and with the software cell^D (both Olympus GmbH, Hamburg, Germany). Subsequently, the positions of the stalked glands (upper or lower leaf surface) were noted and their lengths determined using Fiji/ImageJ [47]. Only stalked glands that were in the focal plane from their base to the head were measured. The obtained values were further analyzed with R-Studio [48]. A Kruskal–Wallis test was used due to the nonparametric measurement series. The Wilcoxon Rrank Sum Test with Holm correction was used as a post-hoc test.

#### 4.2.2. Density Measurements

The number of stalked glands on the upper and lower surfaces of leaves were determined by taking lateral images in the middle of 10 healthy leaves from three *B. gigantea* plants. The lengths of the observed leaf regions were identical during all 10 measurements due to the constant stereo macroscope setup described in Section 4.2.1. All stalked glands that could be recognized on these images were counted and the ratios between their numbers on the upper and lower leaf surfaces were calculated.

### 4.3. Stimulation Experiments and Kinematical Analyses

#### 4.3.1. Stimulation Experiments

Stalked glands situated on the upper surfaces of healthy leaves from two individuals of *B. gigantea*, which were in the focal plane, were observed using the stereo macroscope setup (described in Section 4.2.1) and their lengths were measured. The reaction time, i.e., the time taken by the stalked glands from stimulation until the onset of the respective response movement, as well as the movement duration and general kinematics were determined by recording time-lapse movies (recording speed: one frame per minute) for the following stimulation scenarios.

1. Pure chemical stimulation: Small fish flake food fragments (a few mm in diameter) (TetraMin Flakes, Tetra GmbH, Melle, Germany) were carefully placed on the glue drops of the stalked gland heads using forceps (cf. [11]). Care was taken to deposit the flakes only on the glue drops and to not exert mechanical stimuli on the stalked glands‘ heads and stalk cells. For the experiments, 10 stalked glands from two leaves of *Byblis*-1 and 10 stalked glands from 7 leaves of *Byblis*-2 were used.
2. Pure mechanical stimulation: Stalked glands were stimulated with bursts of five quick consecutive touches with an eyelash (diameter: 80 μm) either on their heads or bases, which took place at intervals of 5 min during a 60 min period each. For the experiments, 10 stalked glands from four leaves of *Byblis*-1 and 10 stalked glands from four leaves of *Byblis*-2 were used.
3. Combined chemical and mechanical stimulation: Fish flake food fragments were placed on the glue drops of stalked gland heads according to the protocol described for pure chemical stimulation. The stalked glands were then tapped following the protocol for pure mechanical stimulation. For the experiments, 10 stalked glands from two leaves of *Byblis*-1 and 10 stalked glands from two leaves of *Byblis*-2 were used.

The measured values were sorted according to the type of stimulus and plant individuum tested. Since the measured values were parametric, one-way ANOVA with Tukey HSD post-hoc tests were used. The stimulation experiments were carried out in the microscopy lab of the Plant Biomechanics Group, Freiburg. For each test, the relative humidity, temperature, and stalked gland length were measured. To evaluate whether possible fluctuations in environmental conditions and differences in stalked gland length affected the reaction time and movement duration, correlation analyses were performed using R.

Stalked glands were also stimulated with a dead aphid (unknown species, collected in the Freiburg Botanical Garden). A fruit fly, representing a relatively large and strong prey animal, was also placed onto a trap leaf to evaluate whether the struggling of the fly can cause damage to the stalked glands.

Furthermore, we carefully attached 17 small Styrofoam beads (diameter: ca. 0.8 mm) to the heads of stalked glands located on leaves from two additional, healthy *B. gigantea* plants. After 6 h, the glands were examined for possible movement reactions. These additional analyses took place in the temperate greenhouse (temperature: 25 °C; relative humidity: 70%) of the Darmstadt Botanical Garden.

#### 4.3.2. Experiments with Stalked Glands on Detached Leaf Pieces

We also tested whether stalked glands situated on cut-off leaf pieces were able to be stimulated and move. For this purpose, five ~2–4 cm long leaf pieces were separated from the plant with a scalpel and the cut surfaces sealed with Vaseline. The pieces were fixed in Petri dishes with a thin wire and weighted with two 10 Euro-Cent pieces. The base of each Petri dish was covered with wet paper towel. The entire dish was then covered with another Petri dish, which was removed only for image recording. Stalked glands were stimulated with fish flake food (as described in Section 4.3.1). Time-lapse movies (recording speed: 1 frame per minute) were recorded using the stereo macroscope setup described in Section 4.2.1. Stimulation was typically performed immediately after leaf separation. In one case, stimulation was performed after three days.

#### 4.3.3. Temperature Dependencies of Reaction Time and Movement Duration

To analyze the influence of temperature on movement, stalked glands situated on intact leaves of a healthy plant were stimulated with fish flake food in both warm (22 °C) and cold (12 °C) temperature regimes in constant chambers of the Bio II/III department of the University of Freiburg (relative humidity ~70%). The plants received artificial light and were acclimatized to the respective conditions overnight prior to the experimental procedure. The responses of the stalked glands to stimulation were recorded with the stereo macroscope setup described in Section 4.2.1 (recording speed: 1 frame per minute). Images were analyzed for reaction time and movement duration with Fiji/ImageJ and compared with R using a *t*-test for significant differences between the temperature regimes.

#### 4.3.4. Analysis of Water Displacement in the Stalk Cell

The movement of the stalked glands is accompanied by the removal of fluid from the stalk cells. Therefore, we additionally tested whether the fluid is (partly) released to the environment or (completely) transported into the basal cells. For this purpose, six trichomes from different leaves of one plant were stimulated with fish flake food (as described in Section 4.3.1). After application of the flake fragments, the respective leaves were transferred into a Petri dish filled with thin paraffin oil. The movements of the stalked glands were recorded with one frame per minute using the stereo macroscope setup mentioned in Section 4.2.1 and the resulting videos were analyzed and the reaction time and movement duration identified with Fiji/ImageJ.

### 4.4. Dehydration and Rehydration Experiments

#### 4.4.1. Dehydration and Rehydration of Non-Stimulated Stalked Glands

Small leaf pieces with non-stimulated stalked glands were cut with a razor blade and treated as follows.

1. Drying in air: A small leaf piece was clamped between two microscope slides. A single, protruding stalked gland was recorded (recording speed: 1 frame per 15 s) with a SZX7 stereo microscope (Olympus GmbH, Hamburg, Germany) equipped with a ColorViewII camera and by using the cell^D software during drying at ambient conditions of ~60% relative humidity and ~26 °C in the microscopy lab of the Plant Biomechanics Group Freiburg;
2. “Drying” in a hypertonic aqueous solution: A small piece of leaf was submerged in saturated NaCl solution (Carl Roth GmbH + Co. KG, Karlsruhe, Germany) in a small watch glass dish. Stalked glands were recorded with the same setup and recording speed as described for the experiment “Drying in air”;
3. Rehydration with distilled water: At the end of the experiment “Drying in a hypertonic aqueous solution”, the NaCl-solution was aspirated by a paper towel and distilled water was carefully added with a pipette. Stalked glands were recorded with the same setup and recording speed as described for the experiment “Drying in air”.

#### 4.4.2. Rehydration of a Chemically Stimulated Stalked Gland

According to [11], stimulated stalked glands (which had already moved) do not regain their initial conditions and cannot become stimulated again. To investigate this further, one stalked gland on a healthy leaf was stimulated with fish flake food (as described in Section 4.3.1). After its movement was finished, remnants of the flake were carefully brushed off with an eyelash and a leaf-piece was cut with a razor blade. The leaf-piece with the stimulated stalked gland, which had moved, was then placed on a small watch glass dish with distilled water. The stalked gland was recorded at 1 frame per minute with the stereo microscope setup described in Section 4.4.1.

#### 4.4.3. Test for Glue Production in Rehydrated Stalked Glands

These tests were carried out on stalked glands on cut leaf pieces situated in a Petri dish with wet paper towel. The stalked glands were stimulated with fish flake food (as described in Section 4.3.1) and the movements were recorded (1 frame/30 min) with the stereo microscope setup described in Section 4.4.1. The Petri dish was then filled with distilled water so that the leaf-piece was completely drowned. The fish food flakes detached from the stalked glands without any further intervention. The water was pipetted off again after 135–140 min and images of the stimulated and now rehydrated trichomes were taken. We waited approximately 14 h for new glue secretion until further images of the stalked glands were taken.

### 4.5. Functional-Morphological Analyses

#### 4.5.1. General Light Microscopy Analyses

Leaves with non-stimulated stalked glands were cut into small pieces (length ~0.5 cm) and fixed in formalin-acetic acid alcohol (FAA: 63% ethanol, 27% distilled water, 5% glacial acetic acid, 5% formalin). Samples were then dehydrated in an isopropanol series (70% overnight; 2 h 90%, deaerated; 30 min 100%; deaerated; 1 h 100%; deaerated) at room temperature. For pre-infiltration, the samples were placed in a 1:1 solution of isopropanol and Technovit 7100 liquid (Heraeus Kulzer GmbH, Hanau, Germany), treated for 10 min in an ultrasonic bath (Emmi-H60, EMAG-AG, Mörfelden-Walldorf, Germany), and left overnight in a desiccator after deaeration. Subsequently, the samples were infiltrated in Technovit 7100 liquid with Technovit 7100 hardener 1 and deaerated for three days in a refrigerator. Technovit 7100 Hardener 2 was added to the infiltration solution for polymerization. Sections (thickness: 5 µm) were produced by using a rotating microtome (Leica, Wetzlar, Germany) and stained for 30 s with 0.05% toluidine blue. Microscopy slides were sealed with Entellan (Merck KGaA, Darmstadt, Germany). Images were acquired using an BX61 microscope with a DP71 camera and cell^P software (Olympus GmbH, Hamburg, Germany).

#### 4.5.2. Scanning Electron Microscopy Analyses

Leaves with stimulated (with fish food flak fragments) and non-stimulated stalked glands were cleaned in water, then dehydrated in a methanol series (30–100% in steps of 10%, acetone; at least 8 h each) and critical-point dried (CPD030, BAL-TEC AG, Balzers, Liechtenstein). After mounting on aluminum stubs with conductive double-sided adhesive tabs (Plano GmbH, Wetzlar, Germany), the samples were sputtered with gold (thickness approx. 10 nm) using a Sputter Coater 108 auto (Cressington Scientific Instruments Ltd., Watford, UK). The LEO 435 VP SEM (LEO Electron Microscopy Ltd., Cambridge, UK) was used.

#### 4.5.3. Determination of the Cellulose Microfibril Angles in Stalk Cells

Cellulose microfibril angles of the stalk cells of 17 stalked glands were determined according to [42]. First, a small piece of leaf with non-stimulated stalked glands was cut and cleaned in distilled water. Afterwards, a thin strip was cut from the leaf-piece with a razor blade and placed on a microscopy slide with distilled water. Then, 10 μL of chlorine-zinc-iodine solution (10 mL distilled water, 6.5 g KI, 1.3 g I, 20 g ZnCl2) was drawn under the coverslip. After one minute, distilled water was drawn under the coverslip and removed with a paper towel on the other side until the staining solution was completely rinsed off. The sample was examined with polarized light using the microscope setup described in Section 4.5.1. Different focal planes were acquired, which were merged into one image afterwards using a Z-projection (maximum intensity) in Fiji/ImageJ and Adobe Photoshop CS6 (Adobe Inc, San José, CA, USA). The cellulose microfibril angles were determined in ImageJ. For this purpose, the trichomes were divided into five sections of equal length. In each section the angles of the stained cellulose microfibrils were determined in relation to the central longitudinal axis of the stalk cell. Statistical analysis of the data was performed in R. First, the angles of the different sections were generally analyzed for significant differences using a Kruskal–Wallis test. Then, a pairwise t-test was performed to specify the differences.

#### 4.5.4. Histological Staining of Stalk Cells

The stalk cells were further characterized histologically via staining protocols according to [49]. For this purpose, small leaf sections with stalked glands were cut and prepared as follows:

1. Cellulose staining: The sample was placed in a 0.01% Calcofluor white solution for 10 min and then rinsed with distilled water. Separated trichomes were placed in distilled water on a slide and viewed and photographed under UV light using the microscope setup described in Section 4.5.1;
2. Cutin staining: The sample was placed in Sudan IV solution (1 g Sudan IV, 100 mL 80% ethanol, 10 mL glycerol) for 30 min and then briefly rinsed with 80% ethanol and thoroughly rinsed with distilled water. The trichomes were fixed in glycerol gelatin on the slide and investigated using the microscope setup described in Section 4.5.1;
3. Pectin staining: A 0.1% ruthenium red solution was applied to the leaf-piece for five minutes and then rinsed twice with distilled water. The stained trichomes were placed in glycerol gelatin on the slide and investigated using the microscope setup described in Section 4.5.1;
4. Lignin staining: The sample was placed in a phloroglucinol solution (10 g phloroglucinol, 100 mL 95% ethanol) for 20 min. Afterwards, the trichomes were placed in 25% hydrochloric acid on the slide and analyzed with a Primo Star microscope equipped with an Axiocam ERc 5s (Zeiss, Wetzlar, Germany).

## Figures and Tables

**Table 1:** Stalked gland densities measured on 10 leaves from three plants, indicated as ratios between the stalked gland numbers on areas identical in dimension on the upper and lower leaf surfaces.

Plant No.	Leaf No.	Ratio	Mean ± SD
*Byblis*-1	1	0.67	0.55 ± 0.15
*Byblis*-1	2	0.38	
*Byblis*-1	3	0.60	
*Byblis*-2	1	0.53	0.45 ± 0.07
*Byblis*-2	2	0.43	
*Byblis*-2	3	0.40	
*Byblis*-3	1	0.81	0.51 ± 0.20
*Byblis*-3	2	0.42	
*Byblis*-3	3	0.43	
*Byblis*-3	4	0.39	
*Byblis* 1–3 (total)			0.51 ± 0.14

## Data Availability

All data are incorporated into the article and its online supplementary material.

## Supplementary Materials

The supporting information can be downloaded at www.mdpi.com/article/10.3390/ijms231911514/s1.
