# Supplementary material for: Chemonastic Stalked Glands in the Carnivorous Rainbow Plant Byblis gigantea LINDL. (Byblidaceae, Lamiales)
Source: Int J Mol Sci. 2022 Sep 29;23(19):11514. doi: 10.3390/ijms231911514 (PMC9569831; doi:10.3390/ijms231911514)
Supplement: Supplementary file 1 [file ijms-23-11514-s001.zip › Supplementary Figures S1-5 and Tables S1-5.pdf]

SUPPLEMENTARY MATERIALS FOR

Chemonastic stalked glands in the carnivorous rainbow plant *Byblis gigantea* LINDL. (Byblidaceae, Lamiales)

S. Poppinga[1,*], N. Knorr[2,3,§], S. Ruppert[2,§], T. Speck[2,3]

[1]Botanical Garden, Department of Biology, Technical University of Darmstadt, Darmstadt, Germany

[2]Botanical Garden, University of Freiburg, Freiburg im Breisgau, Germany

[3]Cluster of Excellence livMatS @ FIT – Freiburg Center for Interactive Materials and Bioinspired Technologies, University of Freiburg, Freiburg im Breisgau, Germany

[§]These authors contributed equally to this study

[*]Corresponding author. E-mail: simon.poppinga@tu-darmstadt.de

Content:

Figures S1-5

Tables S1-5

Figure S1

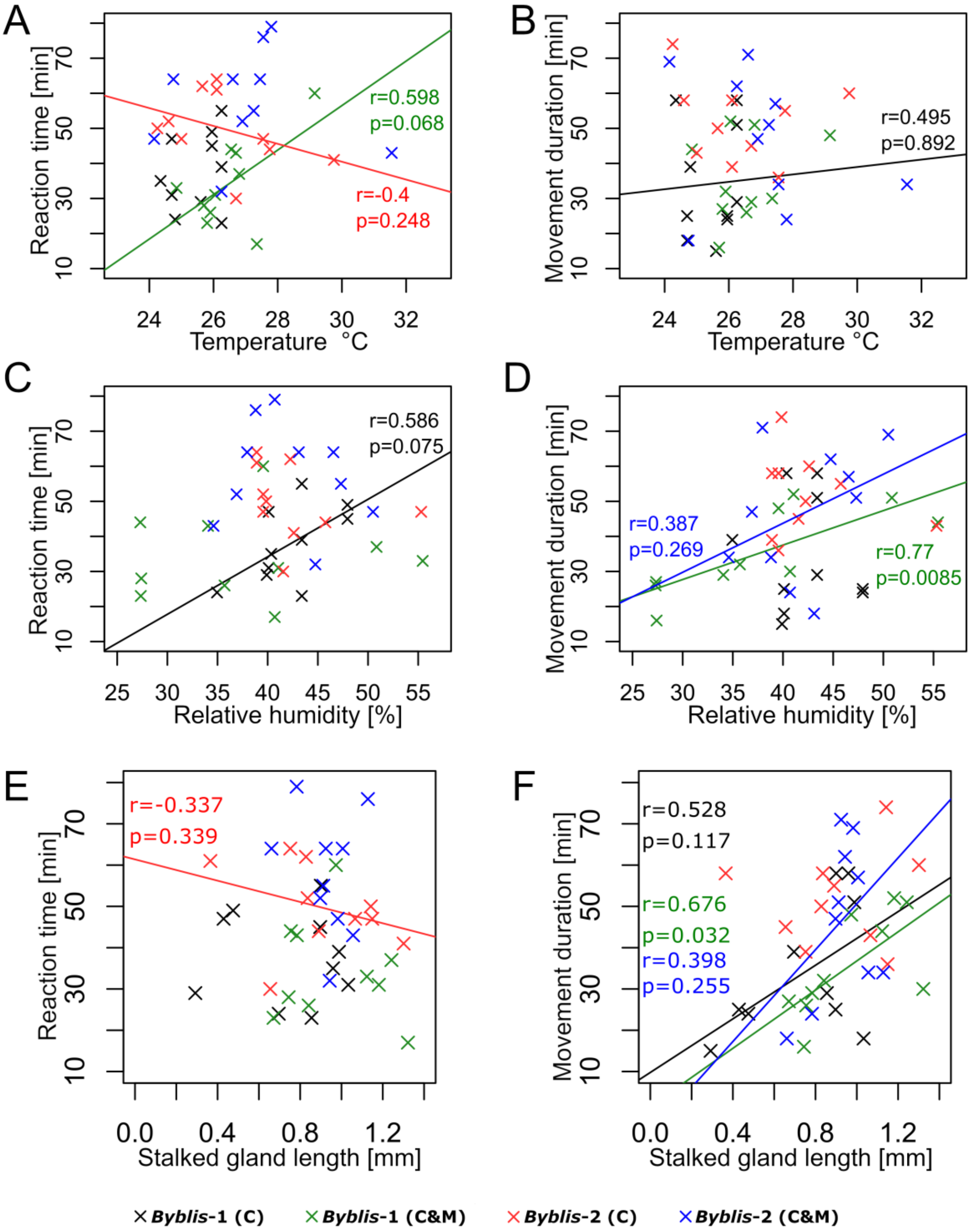

Figure S1. Shows the results from the correlation analyses between (A) reaction time and temperature, (B) movement duration and temperature, (C) reaction time and relative humidity, (D) movement duration and humidity, (E) reaction time and stalked gland length, and (F) movement duration and stalked gland length. Indicated are the two tested plants (*Byblis*-1 and *Byblis*-2) and the stimulation scenarios, i.e., pure chemical (C) and combined chemical and mechanical (C&M) stimulation.

Figure S2

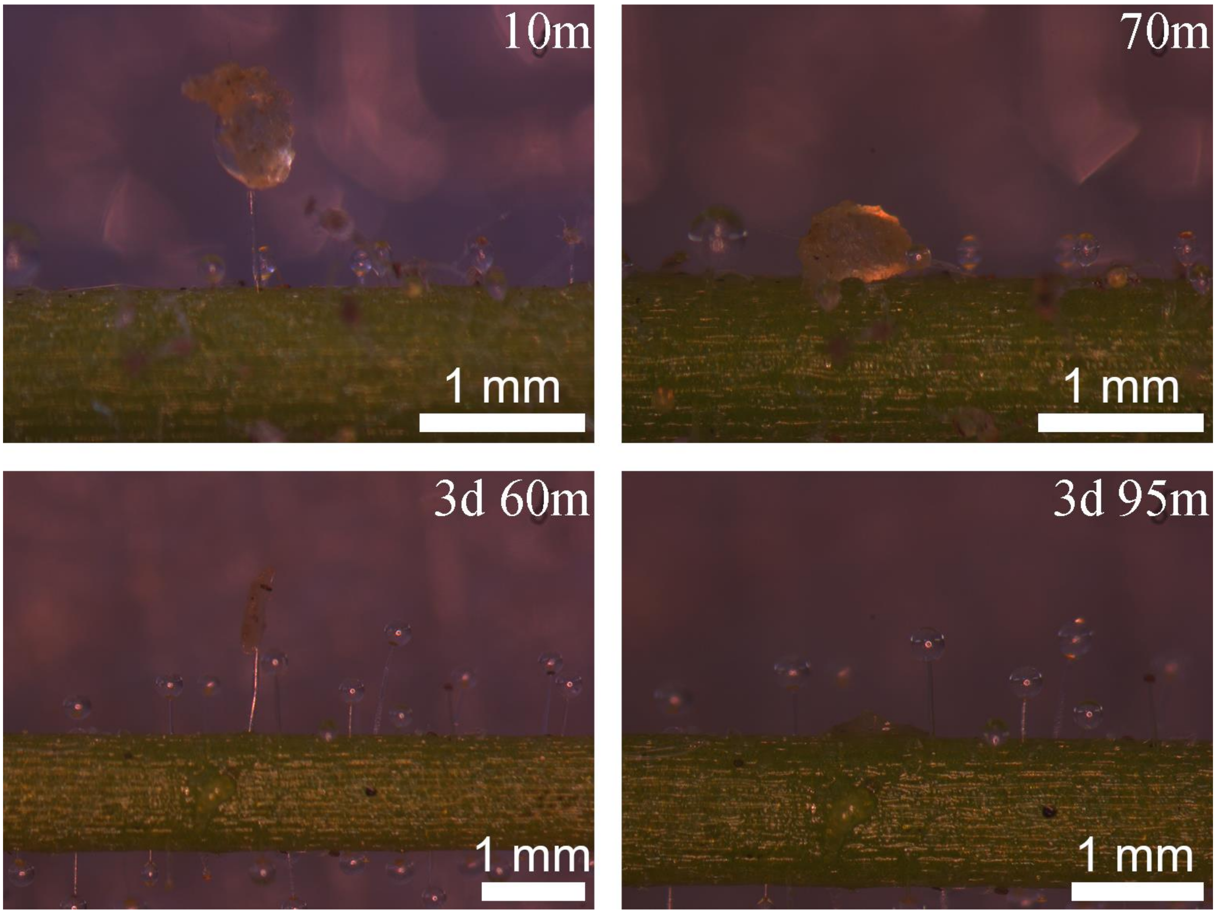

Figure S2. Shows the movement responses of stalked glands situated on the same cut-off leaf piece and stimulated with fish food flake fragments 10 minutes (upper images) and 3 days and 60 minutes (lower images) after detachment of the leaf piece.

Figure S3

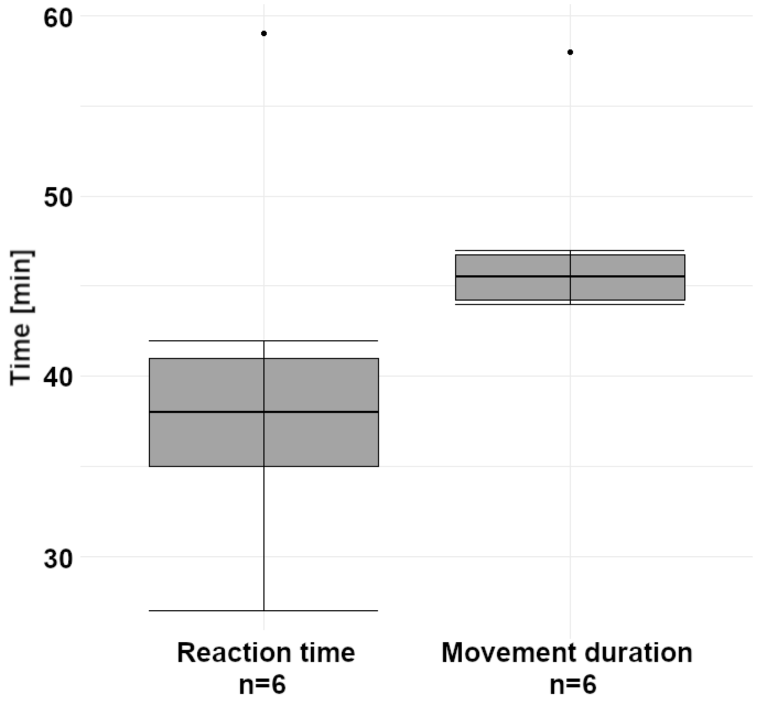

Figure S3. Shows reaction times and movement durations of stalked glands moving in paraffin oil.

Figure S4

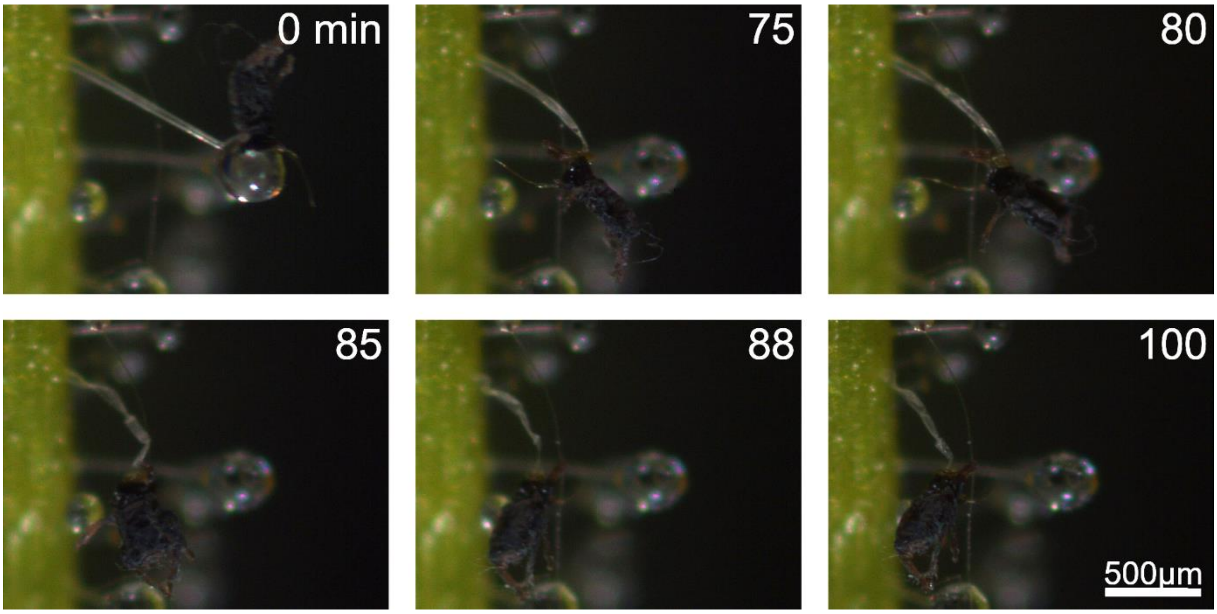

Figure S4. Shows the motion response and respective time scales [min] of a stalked gland stimulated with a dead aphid.

Figure S5

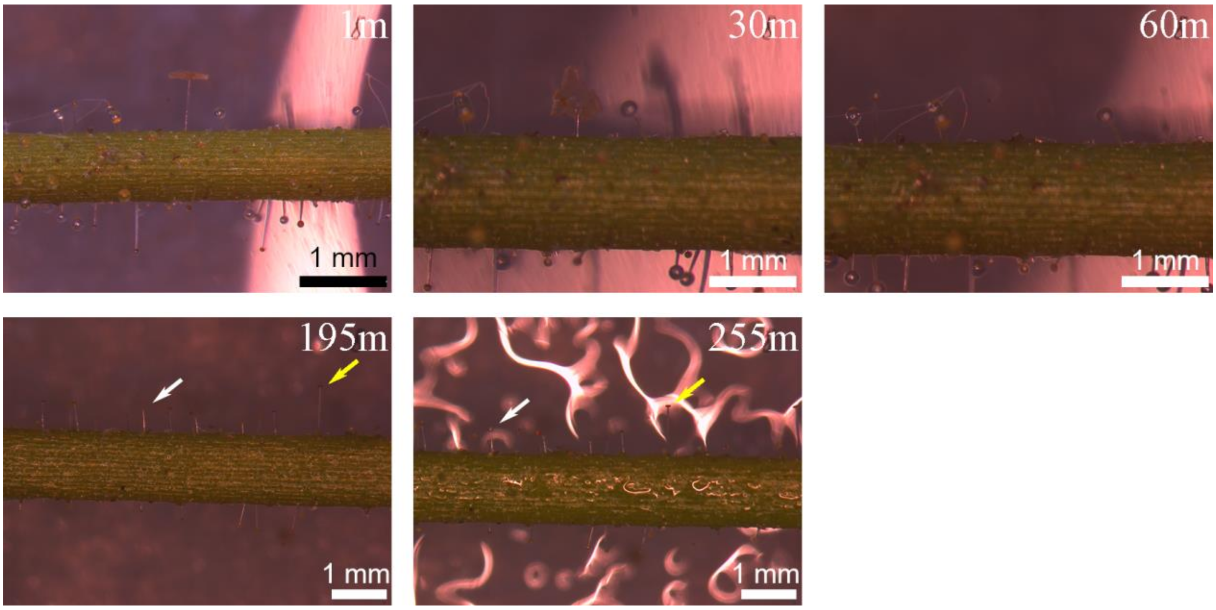

Figure S5. In the three upper images of Figure S5, the movement of a chemically stimulated stalked gland can be seen. The times indicated refer to the moment of stimulation. At minute 195, the rehydrated stalked gland (yellow arrow) and further other stalked glands (one indicated with a white arrow) are indicated. At minute 255, it can be seen that surrounding stalked glands produced glue drops, whereas the stimulated and rehydrated stalked gland did not produce glue.

Table S1

Table S1. Provides the original data for the stalked gland length measurements. US = upper surface of leaf; LS = lower surface of leaf.

Plant	Stalked gland length [mm]	Position on leaf
Byblis-1	0.648	US
Byblis-1	0.265	US
Byblis-1	0.462	US
Byblis-1	0.573	US
Byblis-1	0.603	LS
Byblis-1	0.703	LS
Byblis-1	0.927	LS
Byblis-1	0.494	LS
Byblis-1	1.146	US
Byblis-1	0.522	LS
Byblis-1	0.595	US
Byblis-1	0.935	US
Byblis-1	0.885	LS
Byblis-1	0.354	LS
Byblis-1	0.43	US
Byblis-1	0.636	US
Byblis-1	0.693	LS
Byblis-1	0.514	LS
Byblis-1	1.062	LS
Byblis-1	0.634	US
Byblis-1	0.347	US
Byblis-1	0.588	US
Byblis-1	0.513	US
Byblis-1	0.626	LS
Byblis-1	0.853	LS
Byblis-1	1.154	LS
Byblis-1	0.897	US
Byblis-1	0.475	US
Byblis-1	0.981	US
Byblis-1	0.784	LS
Byblis-1	0.392	LS
Byblis-1	0.378	LS
Byblis-1	0.837	LS
Byblis-1	0.445	LS
Byblis-1	0.588	LS
Byblis-1	0.653	US
Byblis-1	0.637	US
Byblis-1	0.757	LS
Byblis-1	0.573	LS
Byblis-1	1.11	LS

Byblis-1	1.132	LS
Byblis-1	0.347	LS
Byblis-1	0.536	LS
Byblis-1	0.901	LS
Byblis-1	0.354	LS
Byblis-1	0.862	US
Byblis-1	0.385	US
Byblis-1	0.812	US
Byblis-1	0.566	LS
Byblis-1	0.564	LS
Byblis-1	0.678	LS
Byblis-1	0.897	LS
Byblis-1	1.12	US
Byblis-1	0.951	LS
Byblis-1	0.46	LS
Byblis-1	0.574	LS
Byblis-1	0.579	LS
Byblis-1	1.124	US
Byblis-1	0.282	US
Byblis-1	0.291	US
Byblis-1	0.51	US
Byblis-1	0.99	US
Byblis-1	0.598	LS
Byblis-1	0.639	US
Byblis-1	0.569	US
Byblis-1	0.285	US
Byblis-1	0.365	US
Byblis-1	1.059	LS
Byblis-1	0.688	LS
Byblis-1	0.642	LS
Byblis-1	0.698	LS
Byblis-1	0.867	LS
Byblis-1	0.598	US
Byblis-1	0.299	US
Byblis-1	0.688	LS
Byblis-1	0.253	LS
Byblis-1	0.604	LS
Byblis-1	0.752	LS
Byblis-1	0.364	US
Byblis-1	0.421	US
Byblis-1	0.477	US
Byblis-1	0.566	LS
Byblis-1	0.631	LS
Byblis-1	0.257	US
Byblis-1	0.661	US
Byblis-1	0.5	LS
Byblis-1	0.737	LS

Byblis-1	0.309	US
Byblis-1	0.892	LS
Byblis-1	0.853	LS
Byblis-1	1.146	LS
Byblis-1	0.468	LS
Byblis-1	1.027	LS
Byblis-1	1.102	LS
Byblis-1	0.908	LS
Byblis-1	0.931	LS
Byblis-1	1.213	LS
Byblis-1	0.646	LS
Byblis-1	0.613	US
Byblis-1	0.564	US
Byblis-1	0.691	US
Byblis-1	0.438	US
Byblis-1	0.565	LS
Byblis-1	0.923	LS
Byblis-1	0.829	LS
Byblis-1	1.304	LS
Byblis-1	0.936	US
Byblis-1	0.492	US
Byblis-1	0.733	US
Byblis-1	1.182	LS
Byblis-1	0.558	LS
Byblis-1	0.83	LS
Byblis-1	0.722	US
Byblis-1	0.686	US
Byblis-1	0.367	US
Byblis-1	0.845	US
Byblis-1	0.44	LS
Byblis-1	1.084	LS
Byblis-1	0.548	LS
Byblis-1	0.775	LS
Byblis-1	0.375	LS
Byblis-1	0.686	US
Byblis-1	0.944	US
Byblis-1	0.243	US
Byblis-1	0.467	US
Byblis-1	0.902	US
Byblis-1	0.852	LS
Byblis-1	0.92	LS
Byblis-1	0.611	LS
Byblis-1	0.816	LS
Byblis-1	0.93	US
Byblis-1	0.974	US
Byblis-1	0.287	US
Byblis-1	0.989	LS

Byblis-1	0.907	LS
Byblis-1	0.607	LS
Byblis-1	0.847	LS
Byblis-1	0.423	LS
Byblis-1	0.484	LS
Byblis-1	0.393	LS
Byblis-1	0.46	US
Byblis-1	0.486	US
Byblis-1	0.325	US
Byblis-1	0.423	LS
Byblis-1	0.595	LS
Byblis-1	0.324	LS
Byblis-1	0.438	US
Byblis-1	0.362	US
Byblis-1	0.544	US
Byblis-1	0.641	LS
Byblis-1	0.377	LS
Byblis-1	0.55	LS
Byblis-1	0.445	LS
Byblis-1	0.393	US
Byblis-1	0.43	US
Byblis-1	0.399	US
Byblis-1	0.597	LS
Byblis-1	0.437	LS
Byblis-1	0.564	LS
Byblis-1	0.355	LS
Byblis-1	0.437	US
Byblis-1	0.279	US
Byblis-1	0.454	LS
Byblis-1	0.284	LS
Byblis-1	0.626	LS
Byblis-1	0.445	LS
Byblis-2	0.543	US
Byblis-2	0.635	US
Byblis-2	0.486	LS
Byblis-2	0.694	LS
Byblis-2	0.463	LS
Byblis-2	0.41	LS
Byblis-2	0.683	LS
Byblis-2	0.353	LS
Byblis-2	0.491	LS
Byblis-2	0.421	LS
Byblis-2	0.169	US
Byblis-2	0.815	LS
Byblis-2	0.589	LS
Byblis-2	0.847	LS
Byblis-2	0.534	LS

Byblis-2	0.68	US
Byblis-2	0.282	US
Byblis-2	0.436	US
Byblis-2	0.377	LS
Byblis-2	0.377	LS
Byblis-2	1.263	LS
Byblis-2	0.715	US
Byblis-2	0.815	US
Byblis-2	0.769	LS
Byblis-2	0.55	LS
Byblis-2	0.601	LS
Byblis-2	0.464	US
Byblis-2	0.324	US
Byblis-2	0.432	US
Byblis-2	1.057	LS
Byblis-2	0.936	LS
Byblis-2	0.605	LS
Byblis-2	0.596	LS
Byblis-2	0.846	LS
Byblis-2	0.505	LS
Byblis-2	0.988	LS
Byblis-2	0.548	LS
Byblis-2	0.423	LS
Byblis-2	0.427	US
Byblis-2	0.513	US
Byblis-2	0.377	US
Byblis-2	0.452	US
Byblis-2	0.66	LS
Byblis-2	1.044	LS
Byblis-2	0.91	LS
Byblis-2	0.584	LS
Byblis-2	0.542	LS
Byblis-2	0.906	LS
Byblis-2	0.437	LS
Byblis-2	0.692	LS
Byblis-2	0.432	LS
Byblis-2	0.451	LS
Byblis-2	0.368	US
Byblis-2	0.856	LS
Byblis-2	0.321	LS
Byblis-2	0.662	LS
Byblis-2	0.606	US
Byblis-2	0.6	LS
Byblis-2	0.98	LS
Byblis-2	0.589	LS
Byblis-2	0.874	LS
Byblis-2	0.795	LS

Byblis-2	1.178	LS
Byblis-2	0.814	LS
Byblis-2	0.339	LS
Byblis-2	0.537	LS
Byblis-2	0.966	LS
Byblis-2	0.708	US
Byblis-2	0.588	US
Byblis-2	0.483	US
Byblis-2	0.665	US
Byblis-2	0.43	US
Byblis-2	0.305	US
Byblis-2	0.467	LS
Byblis-2	1.193	LS
Byblis-2	0.713	LS
Byblis-2	0.311	LS
Byblis-2	0.575	LS
Byblis-2	0.615	LS
Byblis-2	0.252	US
Byblis-2	0.46	US
Byblis-2	0.437	US
Byblis-2	0.498	US
Byblis-2	0.423	US
Byblis-2	0.476	US
Byblis-2	0.52	US
Byblis-2	0.505	LS
Byblis-2	1.457	LS
Byblis-2	0.655	US
Byblis-2	0.597	US
Byblis-2	0.783	US
Byblis-2	0.524	US
Byblis-2	0.64	US
Byblis-2	0.29	US
Byblis-2	0.773	LS
Byblis-2	0.642	LS
Byblis-2	1.227	LS
Byblis-2	1.062	LS
Byblis-2	1.271	LS
Byblis-2	0.574	US
Byblis-2	0.94	US
Byblis-2	0.832	US
Byblis-2	0.777	US
Byblis-2	0.386	LS
Byblis-2	0.828	LS
Byblis-2	0.35	LS
Byblis-2	0.698	LS
Byblis-2	0.88	LS
Byblis-2	0.761	LS

Byblis-2	0.415	US
Byblis-2	0.443	US
Byblis-2	0.691	US
Byblis-2	0.807	US
Byblis-2	0.729	LS
Byblis-2	0.771	LS
Byblis-2	1.214	LS
Byblis-2	0.399	US
Byblis-2	1.052	US
Byblis-2	0.874	US
Byblis-2	0.498	US
Byblis-2	0.754	LS
Byblis-2	0.671	LS
Byblis-2	1.269	LS
Byblis-2	1.176	LS
Byblis-2	0.725	LS
Byblis-2	1.3	US
Byblis-2	1.1951	US
Byblis-2	0.664	US
Byblis-2	1.394	US
Byblis-2	0.71	US
Byblis-2	0.98	LS
Byblis-2	0.966	LS
Byblis-2	1.022	LS
Byblis-2	1.512	LS
Byblis-2	1.186	LS
Byblis-2	1.078	US
Byblis-2	0.445	US
Byblis-2	0.838	US
Byblis-2	0.666	US
Byblis-2	0.875	US
Byblis-2	0.688	US
Byblis-2	0.808	US
Byblis-2	0.809	US
Byblis-2	1.295	LS
Byblis-2	0.808	LS
Byblis-2	0.902	LS
Byblis-2	0.517	LS
Byblis-2	0.748	US
Byblis-2	0.59	US
Byblis-2	1.271	US
Byblis-2	0.37	US
Byblis-2	0.498	US
Byblis-2	0.447	LS
Byblis-2	0.453	LS
Byblis-2	0.995	LS
Byblis-2	0.597	US

Byblis-2	1.108	US
Byblis-2	0.822	US
Byblis-2	0.641	LS
Byblis-2	1.1	LS
Byblis-2	0.806	LS
Byblis-2	0.787	LS
Byblis-2	0.484	US
Byblis-2	0.475	US
Byblis-2	0.536	US
Byblis-2	0.528	US
Byblis-2	1.364	LS
Byblis-2	0.611	LS
Byblis-2	0.626	LS
Byblis-2	0.801	US
Byblis-2	0.912	US
Byblis-3	0.52	US
Byblis-3	0.46	US
Byblis-3	0.649	US
Byblis-3	0.513	LS
Byblis-3	0.385	LS
Byblis-3	0.731	LS
Byblis-3	1.228	US
Byblis-3	0.696	US
Byblis-3	0.735	LS
Byblis-3	0.47	LS
Byblis-3	0.445	US
Byblis-3	0.386	US
Byblis-3	1.481	LS
Byblis-3	1.161	LS
Byblis-3	1.1951	LS
Byblis-3	0.73	LS
Byblis-3	1.814	US
Byblis-3	1.128	US
Byblis-3	1.463	US
Byblis-3	2.155	LS
Byblis-3	1.448	LS
Byblis-3	0.583	LS
Byblis-3	1.297	US
Byblis-3	1.098	US
Byblis-3	0.477	US
Byblis-3	0.879	US
Byblis-3	2.361	US
Byblis-3	2.024	US
Byblis-3	2.329	US
Byblis-3	2.735	US
Byblis-3	2.157	US
Byblis-3	1.169	US

Byblis-3	0.468	US
Byblis-3	0.784	US
Byblis-3	0.837	US
Byblis-3	0.309	US
Byblis-3	0.324	US
Byblis-3	0.81	LS
Byblis-3	0.409	LS
Byblis-3	0.432	LS
Byblis-3	0.74	LS
Byblis-3	0.679	LS
Byblis-3	0.448	US
Byblis-3	0.686	US
Byblis-3	0.305	US
Byblis-3	0.906	LS
Byblis-3	0.327	LS
Byblis-3	0.618	LS
Byblis-3	0.781	LS
Byblis-3	1.635	LS
Byblis-3	0.972	LS
Byblis-3	0.684	US
Byblis-3	1.201	US

Table S2

Table S2. Provides the original data for the stalked gland density measurements. US = upper surface of leaf; LS = lower surface of leaf.

Plant number	Number of stalked glands	Position on leaf	Leaf number
1	10	US	1
1	15	LS	1
1	5	US	2
1	13	LS	2
1	12	US	3
1	20	LS	3
2	8	US	1
2	15	LS	1
2	6	US	2
2	14	LS	2
2	8	US	3
2	20	LS	3
3	17	US	1
3	21	LS	1
3	10	US	2
3	24	LS	2
3	9	US	3
3	21	LS	3
3	9	US	4
3	21	LS	4

Table S3

Table S3. Provides the original data for the stalked gland reaction times and movement durations, relative humidity and temperature, and respective stalked gland lengths in the three stimulation scenarios. C = chemical stimulation; M = mechanical stimulation; C&M = combined chemical and mechanical stimulation.

Stimulus scenario	Plant number	Leaf number	Stalked gland length [mm]	Reaction time [min]	Movement duration [min]	Relative humidity (minimum-maximum; mean) [%]	Temperature (minimum-maximum; mean) [°C]
C	1	3	0.696	24	39	33.7-36.2; 34.95	24.3-25.3; 24.8
C	1	2	0.292	29	15	38.2-41.6; 39.9	25.4-25.8; 25.6
C	1	3	1.033	31	18	38.5-41.7; 40.1	24.2-25.2; 24.7
C	1	3	0.429	47	25	38.5-41.7; 40.1	24.2-25.2; 24.7
C	1	3	0.959	35	58	37.9-42.8; 40.35	24.1-24.6; 24.35
C	1	2	0.9	55	58	42.2-44.2; 43.4	25.8-26.7; 26.25
C	1	2	0.988	39	51	42.6-44.2; 43.4	25.8-26.7; 26.25
C	1	2	0.855	23	29	42.6-44.2; 43.4	25.8-26.7; 26.25
C	1	3	0.897	45	25	47.2-48.7; 47.95	25.8-26.1; 25.95
C	1	3	0.475	49	24	47.2-48.7; 47.95	25.8-26.1; 25.95
C	2	2	0.828	62	50	39.0-45.5; 42.25	25.2-26.1; 25.65
C	2	2	0.752	64	39	37.1-40.7; 38.9	26.0-26.2; 26.1
C	2	2	0.365	61	58	37.1-40.7; 38.9	26.0-26.2; 26.1
C	2	2	0.836	52	58	38.2-40.9; 39.55	23.9-25.3; 24.6
C	2	6	1.142	50	74	38.6-41.1; 39.85	24.1-24.4; 24.25
C	2	5	1.066	47	43	54.9-55.7; 55.3	24.9-25.1; 25.0
C	2	9	0.89	44	55	45.1-46.4; 45.75	27.7-27.8; 27.75
C	2	10	1.149	47	36	39.0-40.1; 39.55	27.5-27.6; 27.55
C	2	3	0.655	30	45	40.6-42.5; 41.55	26.7-26.7; 26.7
C	2	9	1.301	41	60	40.3-44.9; 42.6	28.5-31.0; 29.75
C&M	1	2	0.755	44	26	26.9-27.7; 27.3	26.3-26.8; 26.55
C&M	1	2	0.744	28	16	27.4-27.4; 27.4	25.7-25.7; 25.7
C&M	1	3	0.671	23	27	27.3-27.4; 27.35	25.8-25.8; 25.8
C&M	1	6	0.841	26	32	34.9-36.5; 35.7	25.5-26.3; 25.9
C&M	1	6	0.784	43	29	33.7-34.4; 34.05	26.5-26.9; 26.7
C&M	1	3	1.181	31	52	40.0-42.1; 41.05	26.0-26.1; 26.05
C&M	1	7	0.973	60	48	38.5-40.6; 39.55	28.3-30.0; 29.15
C&M	1	2	1.322	17	30	40.3-41.1; 40.7	27.3-27.4; 27.35
C&M	1	2	1.123	33	44	55.4-55.5; 55.45	24.5-25.2; 24.85

C&M	1	6	1.242	37	51	50.0-51.7; 50.85	26.6-27.0; 26.8
C&M	2	2	0.897	52	47	36.2-37.6; 36.9	26.9-26.9; 26.9
C&M	2	8	0.924	64	71	37.1-38.8; 37.95	26.4-26.8; 26.6
C&M	2	2	0.982	47	69	49.0-52.0; 50.5	23.2-25.1; 24.15
C&M	2	9	0.943	32	62	44.4-45.1; 44.75	26.2-26.3; 26.25
C&M	2	9	0.661	64	18	42.7-43.5; 43.1	24.4-25.1; 24.75
C&M	2	2	1.056	43	34	29.2-40.0; 34.6	28.5-34.6; 31.55
C&M	2	8	1.006	64	57	46.5-46.6; 46.55	27.3-27.6; 27.45
C&M	2	10	0.912	55	51	47.0-47.6; 47.3	27.2-27.3; 27.25
C&M	2	10	1.128	76	34	37.9-39.7; 38.8	27.4-27.7; 27.55
C&M	2	2	0.783	79	24	40.2-41.2; 40.7	27.8-27.8; 27.8
M	1	3	0.642	no motion	no motion	32.6-34.7; 33.65	25.1-25.2; 25.15
M	1	3	0.463	no motion	no motion	32.6-34.7; 33.65	25.1-25.2; 25.15
M	1	2	0.859	no motion	no motion	31.3-31.8; 31.55	24.-24.8; 24.8
M	1	2	0.571	no motion	no motion	31.3-31.8; 31.55	24.-24.8; 24.8
M	1	2	0.647	no motion	no motion	31.3-31.8; 31.55	24.-24.8; 24.8
M	2	2	0.800	no motion	no motion	36.0-37.5; 36.75	24.7-25.2; 24.95
M	2	2	1.017	no motion	no motion	36.0-37.5; 36.75	24.7-25.2; 24.95
M	2	6	0.651	no motion	no motion	38.4-38.4; 38.4	25.0-25.1; 25.05
M	2	6	0.583	no motion	no motion	38.4-38.4; 38.4	25.0-25.1; 25.05
M	2	6	0.578	no motion	no motion	38.4-38.4; 38.4	25.0-25.1; 25.05

Table S4

Table S4. Provides the original data for the measured reaction times and movement durations in the two temperature regimes, i.e., warm (22 °C) vs. cold (12 °C).

**Reaction time [min] @ 22° C**	**Movement duration [min] @ 22° C**	**Reaction time [min] @ 12° C**	**Movement duration [min] @ 12° C**
40	56	52	68
28	44	54	73
46	42	84	53
34	55	56	89
38	51	63	92
24	42	48	103
50	63	44	82
41	58	38	

Table S5

Table S5. Provides the original data for the cellulose microfibril angle measurements on the five different stalked gland sections.

Stalk cell number	Section 1 [°]	Section 2 [°]	Section 3 [°]	Section 4 [°]	Section 5 [°]
1	42.8	38.5	32.3	31.3	21.8
2	45.2	39.7	34.8	33.9	30.6
3	41.7	36.9	37.9	33.9	27.2
4	33.5	33.7	30.6	--	--
5	41	45.6	28.3	26.1	27.9
6	38.9	37.5	35.3	35.4	26.1
7	37.2	33.8	35.8	28.7	22.6
8	--	--	--	30.7	24.7
9	38.9	36.5	30.4	33.2	25.3
10	36.2	34.9	35.4	32.3	26
11	38.5	37.8	30.5	32.6	18.4
12	37.6	39.5	36.4	34.1	23.8
13	42.9	37.8	32.7	32.1	25
14	40.2	36.8	32	31.3	27.5
15	38	37.5	38.3	33.4	23.3
16	39.4	39.3	41	38.9	26
17	52.9	44.7	43.6	40.6	28.1
18	50.3	42.3	42.9	41.6	32.5
